# Does Participatory Bird Monitoring Provide Accurate Data for Ecological Research? An Experience in Rural Southwestern Mexico

**DOI:** 10.1002/ece3.72237

**Published:** 2025-10-01

**Authors:** Alexis Mendoza‐Lozana, Rubén Ortega‐Álvarez, Adolfo G. Navarro‐Sigüenza, Víctor H. Jiménez‐Arcos, Leopoldo D. Vázquez‐Reyes

**Affiliations:** ^1^ Posgrado en Ciencias Biológicas Universidad Nacional Autónoma de México Mexico City Mexico; ^2^ Facultad de Estudios Superiores Iztacala Universidad Nacional Autónoma de México Tlalnepantla Estado de México Mexico; ^3^ Centro de Investigación en Alimentación y Desarrollo, AC, Subsede Colima, Investigadoras e Investigadores por México de la Secretaría de Ciencia, Humanidades Tecnología e Innovación (Secihti) Colima Mexico; ^4^ Departamento de Biología Evolutiva, Museo de Zoología, Facultad de Ciencias Universidad Nacional Autónoma de México Mexico City Mexico; ^5^ Unidad Multidisciplinaria de Docencia e Investigación Facultad de Ciencias, Campus Juriquilla, Universidad Nacional Autónoma de México Querétaro Mexico; ^6^ Laboratorio de Herpetología Vivario de la FES Iztacala Universidad Nacional Autónoma de México Tlalnepantla Estado de México Mexico

**Keywords:** community‐based monitoring, data quality assessment, Latin America, participatory ornithology, training

## Abstract

The rise of participatory ornithology projects in the Global North has spurred studies aimed at optimizing the quality of data generated by these initiatives to support conservation efforts. However, in Latin America—where these projects are typically developed in collaboration with rural and indigenous communities—evaluations addressing this issue remain scarce. This study analyzes a community‐based bird monitoring project in southwestern Mexico and highlights key aspects for improving data quality. First, five members of a rural community were trained in bird identification and counting. Subsequently, using data generated by professional ornithologists as a reference, the accuracy and bias in the monitoring data were examined to assess bird species richness and community structure across forest and human‐altered habitats. In addition, hierarchical clustering analyses were employed to identify biological traits of species that affect data quality. The community monitoring data demonstrated sufficient quality to detect changes in bird communities resulting from anthropogenic impacts, though there were identifiable biases associated with forest habitat species, migratory species, and species belonging to the families Trochilidae and Tyrannidae. The results suggest that scientific endeavors in community projects should focus on developing adaptive training strategies to enhance monitors' skills in identifying birds and recording their abundance. The evaluation framework proposed in this study offers a valuable collaborative perspective for improving data quality in community monitoring initiatives across Latin America.

## Graphical abstract

Trained local people are capable to conduct bird monitoring and generates useful data for testing ecological hypotheses related to anthropogenic impact. Scientific endeavors based on community projects should develop training strategies aimed at enhancing monitors' skills in both bird identification and abundance recording. It is important to consider the intrinsic biases associated with local perceptions of birds in order to improve data quality in community‐based monitoring projects in Latin America.

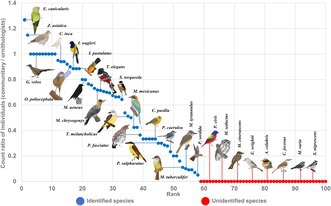

## 1 Introduction

Participatory science—defined as research that involves nonprofessional volunteers in the collection of scientific data—has expanded globally in recent years (Jordan et al. 2015; Aceves‐Bueno et al. 2017). Ornithology is a discipline that has been successfully integrated into participatory projects, aiming to generate ecological data that support conservation actions and biodiversity management (Cooper et al. 2014; Ortega‐Álvarez et al. 2015; Callaghan et al. 2018). Nevertheless, a degree of skepticism persists within the scientific community regarding the quality of data produced through participatory science (Riesch and Potter 2014). This skepticism is based on the notion that data collection requires scientific training and experience; consequently, individuals without such training might produce biased data that do not reliably represent ecological patterns (Riesch and Potter 2014; Theobald et al. 2015). In conjunction with an expansion of participatory ornithology projects in urban settings in the Global North, several studies have sought to evaluate and optimize the quality of data generated by these initiatives (Cooper et al. 2014; Callaghan and Gawlik 2015; Callaghan et al. 2018, 2022). Typically, these evaluations focus on developing statistical tools that mitigate biases associated with volunteer‐generated data, thereby enhancing their utility for addressing specific ecological questions with precision (Bird et al. 2014; Callaghan et al. 2022). This approach has contributed to databases such as eBird providing information of a quality comparable to that produced by professional scientists (Callaghan and Gawlik 2015; Callaghan et al. 2018, 2022).

In contrast to the Global North, participatory ornithology in Latin America primarily emphasizes collaboration with rural and indigenous communities through community‐based monitoring (Constantino et al. 2008, 2012; Ortega‐Álvarez et al. 2015, 2018; Ortega‐Álvarez and Casas 2022). These communities typically inhabit areas of high conservation value but face marginal socioeconomic conditions and rely directly on natural resources to fulfill basic needs such as food, shelter, and employment (Adams et al. 2004). These needs are met through traditional activities that sustain local livelihoods, such as agriculture, livestock rearing, subsistence hunting, and use of forest resources (Agrawal and Gibson 1999; Berkes 2004). When these economic activities are performed without strategies for sustainable management, they can constitute a serious threat to habitat conservation and biological diversity (Agrawal and Gibson 1999; Adams et al. 2004). In this context, community‐based monitoring aims to train local working groups to generate high‐quality data that reliably reflect ecological patterns, thereby supporting autonomous initiatives for biodiversity conservation and sustainable management (Ortega‐Álvarez et al. 2015). These initiatives include the design of agroforestry systems (Vallejo et al. 2015), crop rotation (Beecher et al. 2002), the cultivation of native plants (Burghardt et al. 2009), landscape restoration (Garzón et al. 2020), and the development of avitourism (Lozada‐Ronquillo 2017). Such efforts make it possible to more effectively address the socioecological challenges and needs of local communities (Lentijo and Hostetler 2013; Thomsen et al. 2024). However, to date, no analyses have been undertaken to evaluate the quality of data derived from community‐based ornithological monitoring in Latin America. This contrasts with the Global North, where data quality is a focal topic of research within participatory projects (Jordan et al. 2015; Kosmala et al. 2016).

The rise of citizen science in the Global North has spurred the development of objective criteria for evaluating the quality of data generated by participatory projects (Jordan et al. 2015; Kosmala et al. 2016). Several authors concur that high‐quality data in these initiatives should be characterized by levels of accuracy and bias comparable to those of data produced by professionals (Kosmala et al. 2016). Accuracy refers to the degree to which data correctly represent biological phenomena, while bias denotes the occurrence of systematic error within a dataset. Therefore, the accuracy and bias of volunteer‐generated data are commonly assessed through comparisons with data produced by professional scientists, typically by estimating ecological parameters relevant to biodiversity management, such as species richness and community structure (Callaghan and Gawlik 2015; Kosmala et al. 2016; Callaghan et al. 2022). Analyzing these parameters enables the assessment of the potential of volunteer‐generated data to describe patterns of change in ecological communities associated with human activities. These patterns include reductions in species richness and increases in the dominance of certain species in disturbed environments (Fahrig 2003; Sigel et al. 2010), losses of locally important or conservation‐priority species, and the effects of habitat management on bird diversity (MacGregor‐Fors and Schoundube 2011).

Although volunteer data have proven effective in documenting these ecological patterns at global, regional, and local scales (Dickinson et al. 2010; Callaghan and Gawlik 2015), they tend to exhibit biases more frequently than those generated by scientists (Kosmala et al. 2016). In studies of bird communities, these biases are primarily concentrated in inconspicuous species, species of low abundance, or small‐sized species, which are often more difficult to identify or count accurately (Lewandowski and Specht 2015; Kosmala et al. 2016). Within the context of community‐based monitoring, detecting species prone to bias would contribute to the design of training strategies aimed at enhancing monitors' abilities in both identifying species and recording their abundance, thereby optimizing data quality (Kosmala et al. 2016).

The objective of this study was to evaluate whether community‐based monitoring data are of sufficient quality to describe changes in the species richness and community structure of birds resulting from habitat transformation by human activities. We hypothesized that community monitoring data have sufficient quality to describe ecological changes in bird communities in response to anthropogenic impacts, specifically a reduction in species richness and an increase in community dominance in anthropogenic areas compared to conserved forests. However, we expected that data collected by community monitors would exhibit biases related to biological characteristics, such as conspicuousness, abundance, and body size, that make some birds more difficult to identify and count. These biases are anticipated to be reflected in reduced records of species and abundances by community monitors, resulting in statistically significant differences in the estimates of species richness and community structure when compared to data generated by professional ornithologists.

## 2 Methods

### 2.1 Study Site

**FIGURE 1:**
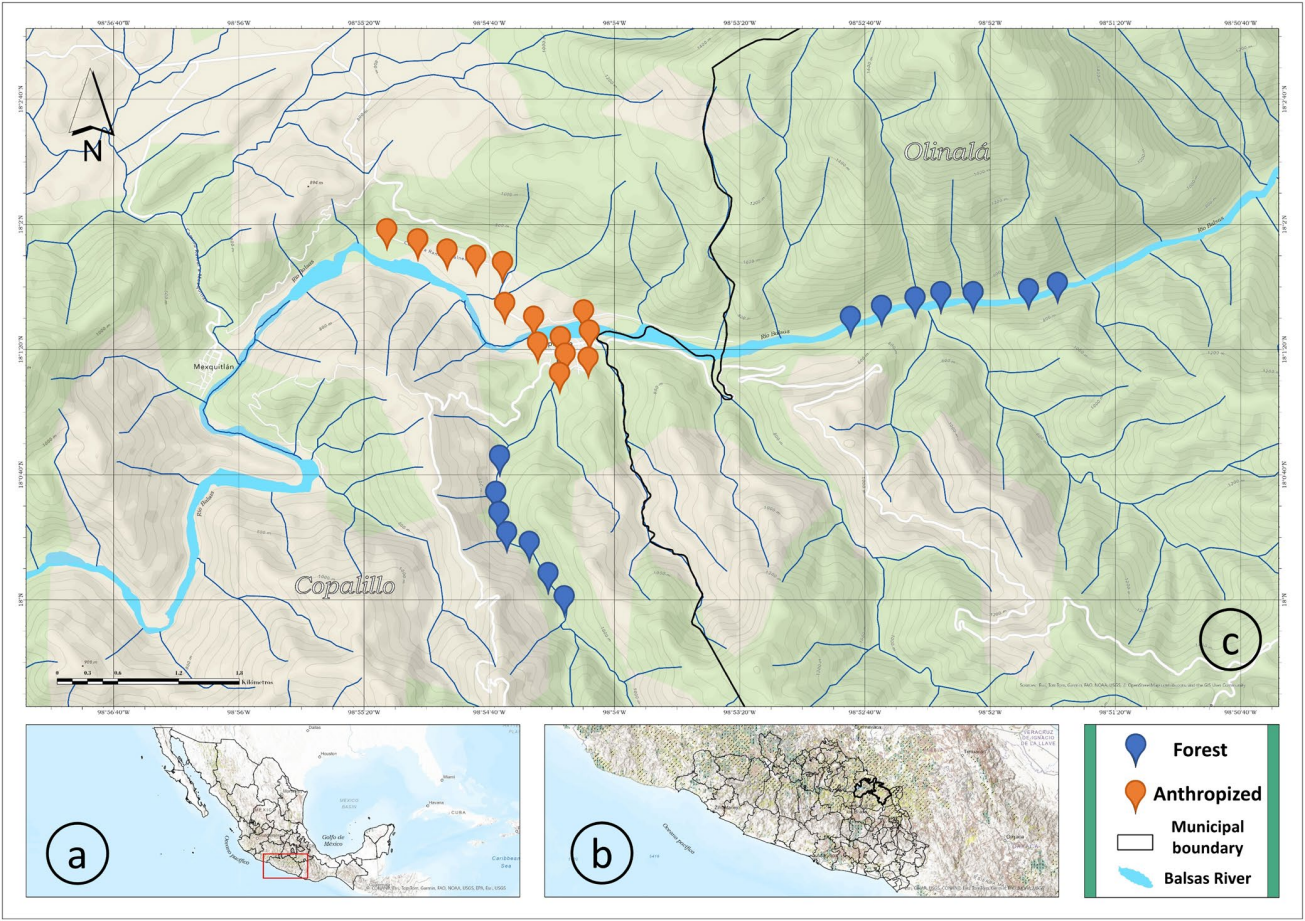
Map of the study site. (a) Location of Guerrero State in southwestern Mexico. (b) Approximate location of the Papalutla community, in northeastern Guerrero. (c) Locations of the point‐count stations in forest habitat (blue labels) and anthropized habitat (orange labels) around the Papalutla community.

The study was conducted in southwestern Mexico, in the rural community of Papalutla (Figure 1), located in northeastern Guerrero (18°01′21″ N; 98°54′15″ W). The dominant vegetation in the area is characterized by tropical deciduous forest distributed across slopes and valleys, with the arboreal layer characterized by *Bursera* spp., *Lysiloma* spp., *Cyrtocarpa procera*, and *Ceiba aesculifolia* (Rzedowski 2006; Vázquez‐Reyes et al. 2018). In ravines and gullies, the vegetation is tropical subdeciduous forest, where the arboreal stratum is represented by *Enterolobium cyclocarpum* , *Ficus* spp., and *Lonchocarpus* spp. Along riverbanks, there are gallery forest elements represented by *Taxodium mucronatum* , *Caesalpinia coriaria* , and *Enterolobium cyclocarpum* (Rzedowski 2006; Vázquez‐Reyes et al. 2018).

The study area is situated in the upper basin of the Balsas River, a region that is globally prioritized for avian conservation due to its high endemic species richness and its contribution to the identity of Mexican biota (BirdLife International 2024a). Human activities primarily include agriculture, livestock rearing, and subsistence hunting (Maldonado et al. 2013). These factors, coupled with the gradual expansion of human settlements, promote the advance of the agricultural frontier and lead to biodiversity loss at both local and landscape scales (Vázquez‐Reyes et al. 2022). Within the study area, three main habitat conditions can be distinguished: forested, secondary growth, and anthropized. The forested habitat comprises areas in which the structure of primary forest vegetation remains intact, with human activities limited to transient passage and occasional hunting. In the secondary growth forest, the structure was altered by agricultural and livestock activities approximately 20 years ago, and since then the tree canopy has partially recovered (Vázquez‐Reyes et al. 2017). The anthropized habitat is characterized by a mosaic of houses, streets, roads, orchards, cultivated fields, and pastures that have replaced the original forest within the community of Papalutla (Vázquez‐Reyes et al. 2022).

### 2.2 Training for Community‐Based Monitoring

Some community members have previously collaborated with academics and civil society organizations on bird conservation projects in Papalutla (Sequi 2016; Vázquez‐Reyes et al. 2018) and have expressed interest in receiving training to serve as community bird monitors (Mendoza‐Lozana 2022). A community‐based monitoring initiative would lay the groundwork for promoting sustainable habitat management and for implementing alternative economic activities based on bird conservation, such as bird tourism (Ortega‐Álvarez et al. 2015; Mendoza‐Lozana 2022). In this context, the rural community of Papalutla has strong potential for the development of sustainable community‐based bird tourism, due to the presence of bird species of interest to birdwatchers and the steady influx of tourists drawn to a local ecotourism‐oriented water park (Mendoza‐Lozana 2022). However, there is a recognized need to strengthen local capacities in bird monitoring and guiding, develop basic infrastructure, such as interpretative trails, and enhance community organization to ensure the long‐term success and sustainability of such an initiative (Mendoza‐Lozana 2022).

Initially, a meeting was convened with community residents to communicate the objectives of this project and to identify individuals interested in training as community bird monitors. The meeting was held at the Papalutla municipal office, selected for its neutral and accessible location to ensure equitable participation conditions. To promote the inclusion of historically underrepresented groups, particularly women (Soares et al. 2023), specific strategies were implemented, including direct invitations, flexible scheduling, and collaboration with local female facilitators. A total of 54 individuals attended the meeting (32 women and 22 men), achieving a higher rate of female participation than typically reported in similar community‐based activities in Latin America (Ortega‐Álvarez et al. 2015; Alejandre‐Ortiz and Ortega‐Álvarez 2024). During the meeting, 10 participants expressed interest in receiving training as community bird monitors, including seven men (average age: 33 years) and 3 women (average age: 15 years). Ultimately, five individuals (all male) completed the training process. While the number of trained participants may appear limited, it should be interpreted in the context of the community's size (approximately 400 inhabitants; Mendoza‐Lozana 2022) and the voluntary nature of the process, which prioritized genuine interest, time availability, and commitment to community‐based bird monitoring.

The training process comprised three stages between March 2023 and March 2024: (1) a theoretical–practical workshop on bird identification and sampling, divided into five sessions lasting 3 h each (Ruiz‐Gutiérrez et al. 2019; Ortega‐Álvarez and Calderón‐Parra 2023); (2) unstructured observation excursions during the first 3 h after dawn to reinforce the theoretical content of the workshop; and (3) bird sampling using fixed‐radius point counts (Ralph et al. 1996).

### 2.3 Bird Sampling

To characterize the bird communities, a total of six sampling events were conducted at bimonthly intervals between April 2023 and March 2024, considering two habitat types: forested and anthropized (Figure 1). In each habitat type, 14 fixed‐radius point count stations were established, each with a radius of 30 m. The stations were separated by at least 200 m to ensure spatial independence among observations (Ralph et al. 1996). During each sampling event, a total of 28 point‐count stations were surveyed (14 per habitat type). Since seven stations were visited per day, each event was conducted over four consecutive days. Surveys were performed during the first 3 h after sunrise, allotting 10 min for observation at each point. During these sessions, the species identity and the number of individuals observed and heard were recorded (Ralph et al. 1996). In total, 168 point‐count stations were sampled: 84 in the forested habitat and 84 in the anthropized habitat.

Sampling was conducted by two groups. The first group, hereafter referred to as “ornithologists,” consisted of professional biologists, including two members (AML and LDVR) with academic training and field experience in ornithology spanning 5 and 17 years, respectively. The second group, hereafter referred to as “community monitors,” was composed of five community members: one secondary education student, one exclusively engaged in agriculture, and three who combine agricultural activities with subsistence hunting. The community monitor team had an average age of 34 years and an average of 9 years of formal education. None of the participants had prior experience with formal bird monitoring activities. In the past, some community monitors had occasionally captured birds for sale as a source of income.

To control the spatial and temporal variation of bird communities, surveys were conducted simultaneously by ornithologists and community monitors, who visited the same point‐count stations on the same day and at the same time. This design also helped control for potential short‐term climatic variations that could affect bird activity and detection. Upon arriving at each point, observers waited 5 min before beginning the count, allowing bird activity to return to normal following any disturbance caused by human presence. During the sampling period, communication between the two groups was limited solely to coordinating the simultaneous start time; any exchange of information regarding bird identification or counting was restricted to members within each group. This approach minimized potential biases related to group size and ensured consistent conditions across all surveys. Consequently, both groups had the same probability of detecting and recording the species present and their abundances at each point‐count station. See Appendix S5 for a detailed summary of the simultaneous surveys between ornithologists and community monitors.

### 2.4 Data Analysis

#### 2.4.1 Species Richness and Community Structure of Birds

To assess differences in species richness between habitat types and sampling groups, we constructed individual‐based rarefaction curves using the *iNext* package in R (Chao et al. 2014; Hsieh et al. 2016). Curves were computed from 500 bootstrap replicates to estimate richness and its 84% and 95% confidence intervals (Colwell et al. 2012; Payton et al. 2003), standardizing comparisons to the minimum observed sample size (number of individuals). Differences were assessed using two complementary approaches. First, a difference was considered significant when 84% confidence intervals did not overlap at the standardized sample size (Payton et al. 2003). Second, we conducted pointwise asymptotic *Z*‐tests on individual‐based rarefaction curves to compare species richness between each pair of communities. These tests were applied at every sample size (*m*) automatically determined by the *iNext* package, which selects the informative points common to all curves based on the data structure (Hsieh et al. 2016). For each *m*, we extracted the estimated species richness and its standard error from 500 bootstrap replicates. At each point, we calculated a *Z* statistic by dividing the difference in richness estimates by the combined standard error—computed as the square root of the sum of the variances of both estimates—and assumed approximate normality under the central limit theorem (Davison and Hinkley 1997; Chao et al. 2014). We considered differences statistically significant at *α* = 0.05 when the absolute *Z* value exceeded 1.96.

To examine differences in the structure of bird communities between habitat types and sampling groups, we applied complementary metrics capturing effective diversity, point dominance, and abundance inequality. We estimated Hill numbers (*q* = 1 and *q* = 2; Jost 2006; Chao et al. 2014), representing effective diversity with sensitivity to common species (*q* = 1) and to dominant species (*q* = 2), respectively. Estimates were obtained using the *iNext* package in R (Hsieh et al. 2016), based on absolute abundances and 500 bootstrap replications. Confidence intervals were set at 84%, following Payton et al. (2003), to allow visual inference of statistical significance based on interval overlap. Point dominance was estimated using the Berger–Parker index, which expresses the proportion of individuals of the most abundant species relative to the total number of individuals in the community (Magurran 2003). Abundance inequality was calculated with the Gini coefficient using the *ineq* package (R Core Team 2025). This coefficient ranges from 0 to 1, with values close to 0 indicating an even distribution of individuals among species, and values near 1 reflecting a strong concentration of abundance in a few species (Restrepo‐Sierra et al. 2024). As a complementary analysis, rank–abundance curves were constructed following Magurran (2003), with abundances expressed as the mean number of individuals per species per count point. Differences in curve slopes, interpreted as indicators of relative evenness, were evaluated using log‐linear regression models (ANCOVA) implemented in PAST (Hammer and Harper 2001). Since the data did not fully meet parametric assumptions, this analysis was interpreted qualitatively as visual support for observed patterns.

#### 2.4.2 Quality of Data Generated by Community Monitors

To evaluate the quality of monitoring data used to document changes in bird communities due to human‐induced habitat transformation, the species richness and community structure patterns generated by community monitors were compared with those produced by ornithologists. Additionally, estimates of species richness and community structure were compared between sampling groups by separately considering the birds recorded in the forested habitat and those in the anthropized habitat.

Two additional variables were defined to assess the quality of the community monitoring data (Kosmala et al. 2016). The degree of accuracy associated with the estimation of species richness was represented by the variable “Identified by Community Monitors.” This variable comprised two mutually exclusive categories: Species identified and species not identified by community monitors. Species that were identified refer to those reported by community monitors during the point counts, whereas unidentified species refer to those recorded by ornithologists but not by community monitors. To represent the level of accuracy in the abundance estimates reported by the community monitoring group, we used the variable “Ratio of observed counts.” This numerical variable was calculated as the ratio of the total number of individuals recorded by the community monitors to the total number of individuals recorded by the ornithologists for each species. Values approaching one indicate highly accurate abundance estimates for a species, values near zero indicate that the community monitors counted fewer individuals than the ornithologists, while values greater than one suggest that community monitors recorded more individuals than the ornithologists for that species.

#### 2.4.3 Improvements in Community Monitoring Data Quality Associated With Accumulated Field Experience

To evaluate potential improvements in data quality generated by the community monitoring group as a function of accumulated field experience, we analyzed its relationship with sampling effort. Sampling effort was defined as the sequence of visits to the 168 point‐count sites included in the study and was used as the independent variable. The dependent variables were “Identified by community monitors” and “Ratio of observed counts.” For operationalization, “Identified by Community Monitors” was calculated as the proportion of species identified by community monitors relative to those recorded by ornithologists at each point‐count site. Similarly, “Ratio of observed counts” was defined as the proportion of individuals recorded by community monitors relative to the total observed by ornithologists per point count.

Since both dependent variables are proportions bounded within the (0,1) interval, beta regression models with logit link were employed, which are appropriate for this type of distribution (Ferrari and Cribari‐Neto 2004). A separate model was fitted for each variable to independently assess their relationship with accumulated sampling effort. Model performance was satisfactory, with pseudo‐*R* ^2^ values of 0.55 for species identification and 0.47 for individual counts, along with high precision parameters (Φ ≈ 90), indicating low dispersion and strong explanatory power (Ferrari and Cribari‐Neto 2004). All analyses were conducted using the *betareg* package in the *R* programming language (Cribari‐Neto and Zeileis 2010).

#### 2.4.4 Biological Traits Related to the Quality of Community Monitoring Data

To determine potential biases affecting the quality of community monitoring data, we considered biological characteristics of birds that could increase the difficulty of accurately identifying them and recording their abundance (Ortega‐Álvarez and Casas 2023; Santangeli et al. 2023). We examined eight variables: recorded abundance, vocal activity, color, conspicuousness, residency status, taxonomic family, habitat, and size (see Table 1 for detailed descriptions).

**TABLE 1:** Description of the biological traits analyzed.

Biological traits	Type of variable	Categories and specifications
Abundance	Numerical	The total number of individuals recorded by professional ornithologists for each species during bird counts
Vocalization	Categorical	*High*: Prolonged vocalizations, very loud volume, or sounds that are highly familiar to local residents *Medium*: Common vocalizations typical of species *Low*: Birds that vocalize infrequently, have low‐volume calls, or are not easily recognized by local residents
Color	Numerical	Measure of color complexity for a bird. Calculated as the chromatic distance between the hues present in a given species and the global average color observed across all bird species (e.g., brown, gray). Birds exhibiting extreme tones that significantly differ from the average colors have high scores, denoting their visual prominence
Conspicuousness	Categorical	*Conspicuous*: Highly noticeable birds; frequently seen or heard in open habitats *Inconspicuous*: Shy birds; usually inhabit dense vegetation and are not easily detected
Residence status	Categorical	*Resident*: The species inhabits the region year‐round *Migratory*: The species is present in the region only during summer or winter
Family	Categorical	Taxonomic family of the species
Habitat	Categorical	*Forest*: The species was recorded exclusively in forest habitat during surveys *Both*: The species was recorded in both forest and anthropized habitats *Anthropized*: The species was recorded exclusively in anthropized habitat during surveys
Mass	Numerical	Species mass in grams

*Note:* The vocal activity and conspicuousness categories proposed by Ortega‐Álvarez and Casas (2023) were employed. Color was based on the estimates provided by Santangeli et al. (2023). The residency status was defined according to the categories of Berlanga et al. (2008). The taxonomic family was determined following Chesser et al. (2023), and the species' mass was established based on Tobias et al. (2022).

We then conducted hierarchical clustering analyses using the R package *ClustOfVar* (Chavent et al. 2012) to identify which of the bird traits were associated with the quality of the data generated by community monitors. We used an agglomerative hierarchical clustering algorithm that can simultaneously include both numerical and categorical variables. This approach is based on principal components analysis for qualitative and quantitative variables (Kiers 1991). Variables were considered homogeneous when they exhibited strong interrelationships, as measured by the Pearson correlation coefficient for quantitative variables and by the correlation ratio for qualitative variables (Chavent et al. 2012). To further elucidate the determinants affecting data quality, we grouped the biological traits of the species with the variable “Identified by Community Monitors” to identify traits that influenced species recognition and therefore species richness. Similarly, to identify traits that affected the accuracy of bird abundance estimates, the biological traits were grouped with the variable “Ratio of observed counts.”

Based on the patterns identified through hierarchical clustering analysis, statistical tests were selected according to variable type to assess the significance and magnitude of associations between biological traits and variables related to data quality. For relationships between categorical variables, chi‐squared tests of independence were applied, complemented by Cramér's *V* as a measure of effect size. To compare continuous variables across categorical groups, analyses of variance (ANOVA) were conducted and partial eta squared (*η* ^2^) was calculated to estimate the proportion of variance explained by each trait. All analyses were performed in R 4.5.1 (R Core Team 2025), using the packages stats and rcompanion. The threshold for statistical significance was set at *α* = 0.05.

Once the bird traits significantly associated with the quality of community monitoring data had been identified, we selected the categories within each trait that could induce biases in identification and abundance. Species belonging to these categories were then removed from the datasets generated by both the ornithologists and the community monitors, and the rarefaction analyses and rank–abundance curve evaluations were repeated to determine whether this resulted in more similar ecological parameters between community monitors and ornithologists. This procedure allowed us to determine whether these species significantly biased the community monitoring data for the estimation of species richness and community structure (Appendix S6).

## 3 Results

A total of 100 bird species were recorded by the ornithologists, distributed among 14 orders, 33 families, and 75 genera (Appendix S7). Community monitors recorded 58 of these species and no additional species that had not been recorded by the ornithologists.

### 3.1 Quality of Data Generated by Community Monitors

Both sampling groups revealed consistent ecological patterns when comparing species richness and community structure of birds between habitat types, although the estimated values differed between groups.

### 3.2 Patterns of Species Richness and Community Structure Reported by Each Sampling Group

**FIGURE 2:**
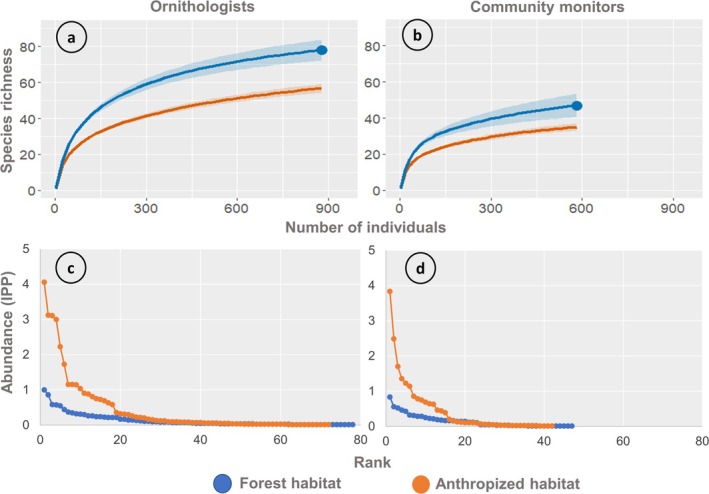
Patterns of species richness and community structure reported by each sampling group. (a) Species richness estimated by ornithologists for forest and anthropized habitats (879 individuals; blue dot). (b) Species richness estimated by community monitors for forest and anthropized habitats (581 individuals; blue dot). (c) Rank–abundance curves estimated by ornithologists for forest and anthropized habitats. (d) Rank–abundance curves estimated by community monitors for forest and anthropized habitats. Shaded area, 95% confidence intervals; IPP, individuals per point count.

According to the rarefaction analysis, both ornithologists and community monitors detected higher species richness in the forested habitat than in the anthropized habitat (Figure 2). The ornithologists recorded 78 ± 6 species in the forested habitat and 57 ± 3 species in the anthropized habitat, with nonoverlapping 84% confidence intervals (Figure 2a). Pointwise comparisons confirmed significantly higher richness in the forested habitat (*Z* > 4.0 across all evaluated sample sizes, *p* < 0.00001; Appendix S8). In a similar trend, the community monitors estimated 47 ± 5 species in the forested habitat and 35 ± 2 species detected in the anthropized habitat—with nonoverlapping confidence intervals (Figure 2b). In this case, significant differences were detected at all evaluated points, with consistently higher richness in the forested habitat (*Z* > 2.59, *p* < 0.00001; Appendix S8).

Both sampling groups exhibited differences in the structure of bird communities between forested and anthropized habitats. Data collected by ornithologists indicated higher effective diversity in forest habitat, both for common species (Hill *q* = 1: 39.50 vs. 24.70) and dominant species (Hill *q* = 2: 26.52 vs. 16.50). The nonoverlap 84% CIs indicate statistically significant differences (Table 2). Additionally, forest habitat exhibited lower point dominance (Berger–Parker: 0.096 vs. 0.146) and reduced inequality in abundance distribution (Gini: 0.616 vs. 0.702) compared to anthropized habitat. Rank–abundance curves supported this pattern, showing steeper slopes in anthropogenic areas (*F*_1,154_ = 34.21, *p* < 0.001), reflecting lower evenness and a higher concentration of individuals in a few dominant species (Figure 2c). Consistently, data gathered by community monitors also demonstrated greater effective diversity in forest habitat, both for common species (Hill *q* = 1: 25.91 vs. 16.60) and dominant species (Hill *q* = 2: 19.34 vs. 11.18). Again, nonoverlapping 84% CIs indicated significant differences between habitat types (Table 2). Forested areas also showed lower point dominance (Berger–Parker: 0.120 vs. 0.202) and reduced inequality in abundance distribution (Gini: 0.585 vs. 0.741), indicating a more even community structure. Rank–abundance curves reinforced this trend, with gentler slopes observed in forest habitat (*F*_1,92_ = 22.39, *p* < 0.001; Figure 2d).

**TABLE 2:** Community structure metrics estimated across habitat types and sampling groups.

Sampling group/habitat status	Habitat status/sampling group	Hill *q* = 1 [84% CI]	Hill *q* = 2 [84% CI]	Berger–Parker	Gini
Ornithologists	Forest	39.53 [37.56–41.51]	26.52 [24.66–28.37]	0.096	0.616
	Anthropized	24.68 [23.94–25.42]	16.42 [15.84–16.99]	0.146	0.702
Community monitors	Forest	25.91 [24.54–27.29]	19.34 [17.99–20.68]	0.120	0.585
	Anthropized	16.56 [15.92–17.20]	11.18 [10.60–11.77]	0.202	0.741
Forest habitat	Ornithologists	39.53 [37.60–41.47]	26.52 [24.69–28.35]	0.096	0.616
	Community monitors	25.91 [24.50–27.33]	19.34 [17.95–20.73]	0.120	0.585
Anthropized	Ornithologists	24.68 [23.95–25.42]	16.42 [15.83–17.00]	0.146	0.702
	Community monitors	16.56 [15.94–17.18]	11.18 [10.59–11.77]	0.202	0.741
Comparisons of community structure excluding species from the families Trochilidae and Tyrannidae, as well as migratory species					
Forest habitat	Ornithologists	22.34 [21.04–23.65]	15.46 [14.23–16.70]	0.140	0.596
	Community monitors	18.98 [17.91–20.05]	14.25 [13.13–15.37]	0.152	0.609
Anthropized	Ornithologists	15.47 [14.96–15.98]	11.23 [10.78–11.68]	0.171	0.704
	Community monitors	15.11 [14.61–15.61]	10.99 [10.51–11.46]	0.180	0.709

*Note:* Overlapping 84% confidence intervals for effective diversity estimates indicate a lack of significant differences between comparisons.

### 3.3 Values of Species Richness and Community Structure Estimated for Each Habitat Type by Sampling Group

**FIGURE 3:**
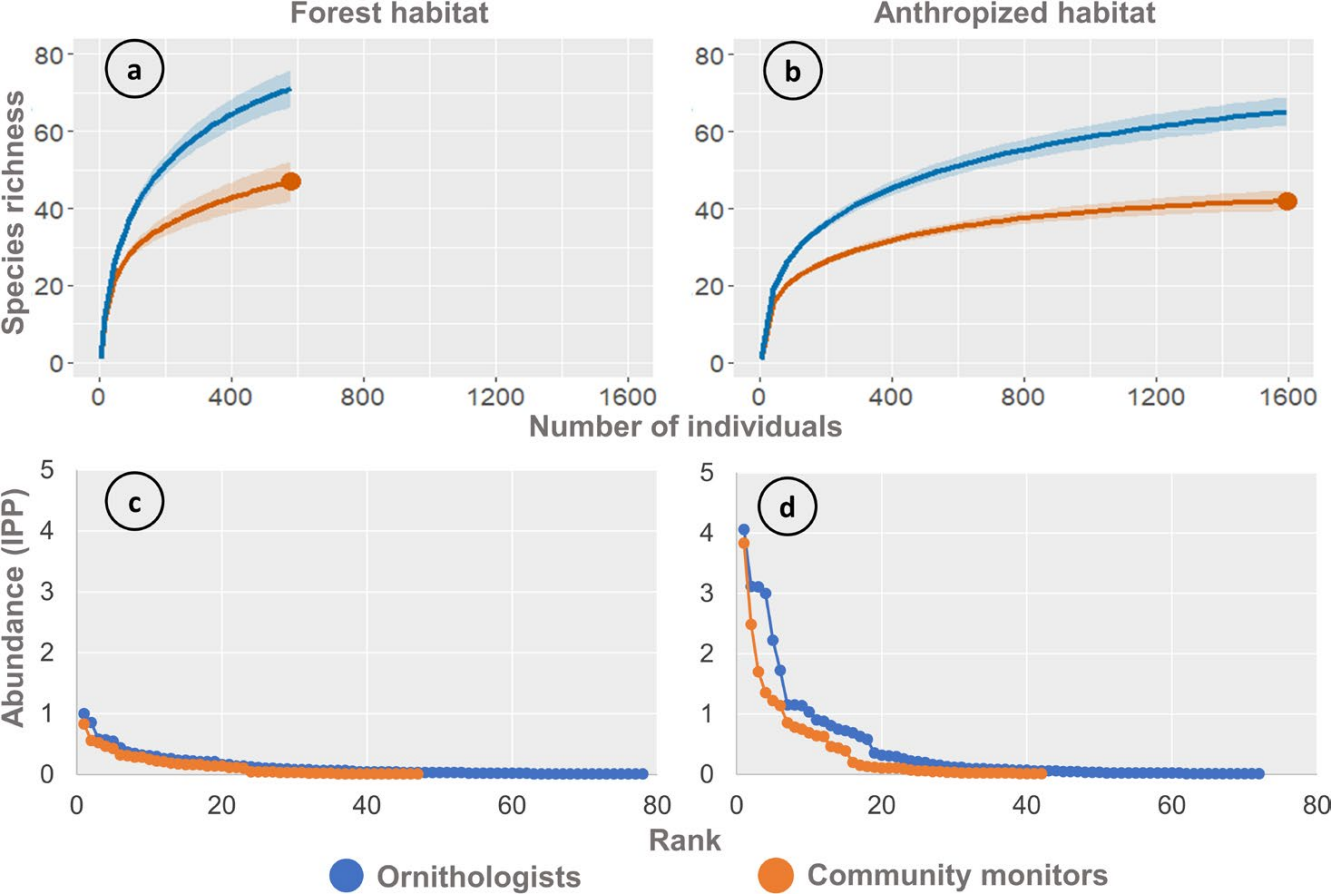
Values of species richness and community structure estimated for each habitat type by sampling group. (a) Species richness estimated for forest habitat by both ornithologists and community monitors (592 individuals; orange dot). (b) Species richness estimated for anthropized habitat by both ornithologists and community monitors (1470 individuals; orange dot). (c) Rank–abundance curves estimated for forest habitat by both ornithologists and community monitors. (d) Rank–abundance curves estimated for anthropized habitat by both ornithologists and community monitors. Shaded area, 95% confidence intervals; IPP, individuals per point count.

**FIGURE 4:**
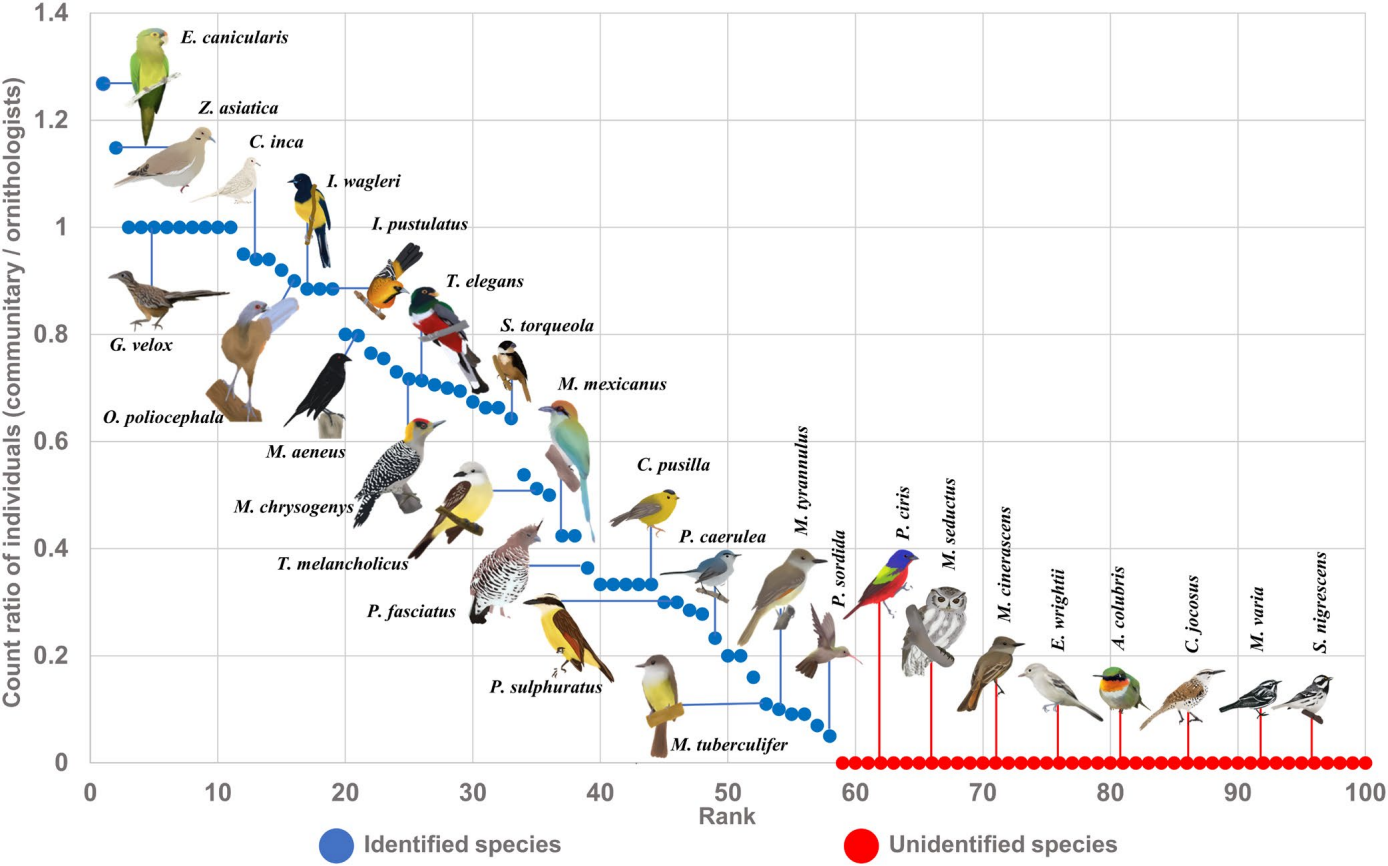
Graphical representation of the variables used to assess the quality of community monitoring data. *Identified by Community Monitors variable*: Blue = species identified by community monitors; Red = species not identified by community monitors. *Ratio of observed counts variable: Y*‐axis = Ratio of the total number of individuals recorded by community monitors to the total number recorded by ornithologists for each species. *X*‐axis = Ordered from left to right, from highest to lowest accuracy in the abundance estimates reported by community monitors. *Illustrations by Montserrat Serra Rojas de la Barrera*.

The species richness values estimated by community monitors were significantly lower than those estimated by ornithologists (Figure 3). In the forest habitat, community monitors estimated a taxonomic richness of 47 ± 6 species, which is lower than the 71 ± 5 species estimated based on ornithologists' data (Figure 3a). Differences were significant across all sampling levels (*Z* > 8.7, *p* < 0.00001; Appendix S8). Similarly, rarefaction analysis of community monitor data yielded an estimate of 42 ± 3 species for the anthropized habitat, compared to an estimate of 65 ± 3 species derived from ornithologists' data (Figure 3b). Pointwise comparisons indicated consistently higher richness in the ornithologists' data across all evaluated sampling levels (*Z* > 8.7, *p* < 0.00001; Appendix S8). The differences in species richness between the two sampling groups were further supported by nonoverlapping 84% confidence intervals. For a detailed list of the species omitted by community monitors, see Figure 4 and Appendix S7.

Contrasts in community structure were observed between data generated by ornithologists and community monitors. In forest habitats, ornithologists recorded higher effective diversity for both common species (Hill *q* = 1: 39.50 vs. 26.00) and dominant species (Hill *q* = 2: 25.30 vs. 24.10), with 84% confidence intervals not overlapping (Table 2). Ornithologists also reported lower point dominance (Berger–Parker = 0.096 vs. 0.120) but greater inequality in abundance distribution (Gini = 0.616 vs. 0.585) compared with community monitors. Slopes of the rank–abundance curves did not differ significantly between sampling groups in forest habitats (*F*_1,154_ = 2.38, *p* > 0.1; Figure 3c). In anthropized habitats, ornithologists again recorded higher effective diversity for both common species (Hill *q* = 1: 24.68 vs. 16.56) and dominant species (Hill *q* = 2: 16.42 vs. 11.18), with 84% confidence intervals showing no overlap (Table 2). In this habitat, ornithologists showed lower point dominance (Berger–Parker = 0.146 vs. 0.202) and lower inequality in abundance distribution (Gini = 0.702 vs. 0.741) relative to community monitors. This pattern was consistent with the rank–abundance curves, whose slopes differed significantly between sampling groups (*F*_1,154_ = 4.61, *p <* 0.01), with community monitoring data showing a stronger dominance effect within the bird community (Figure 3d).

### 3.4 Improvements in Community Monitoring Data Quality Associated With Accumulated Field Experience

Beta regression models revealed a positive and statistically significant effect of accumulated sampling effort on both data quality metrics. For the variable “Identified by Community Monitors,” increased effort was associated with a higher proportion of species identified by community monitors relative to those recorded by ornithologists (*β* = 0.0052, *p* < 0.001). Similarly, for “Ratio of Observed Counts,” a positive relationship was observed between accumulated effort and the proportion of individuals recorded by community monitors compared to ornithologist counts (*β* = 0.0044, *p* < 0.01). These results indicate a progressive and consistent improvement in the quality of data generated by the community monitoring group as field experience increases (Appendix S9).

### 3.5 Biological Traits Associated With the Quality of Community Monitoring Data

**FIGURE 5:**
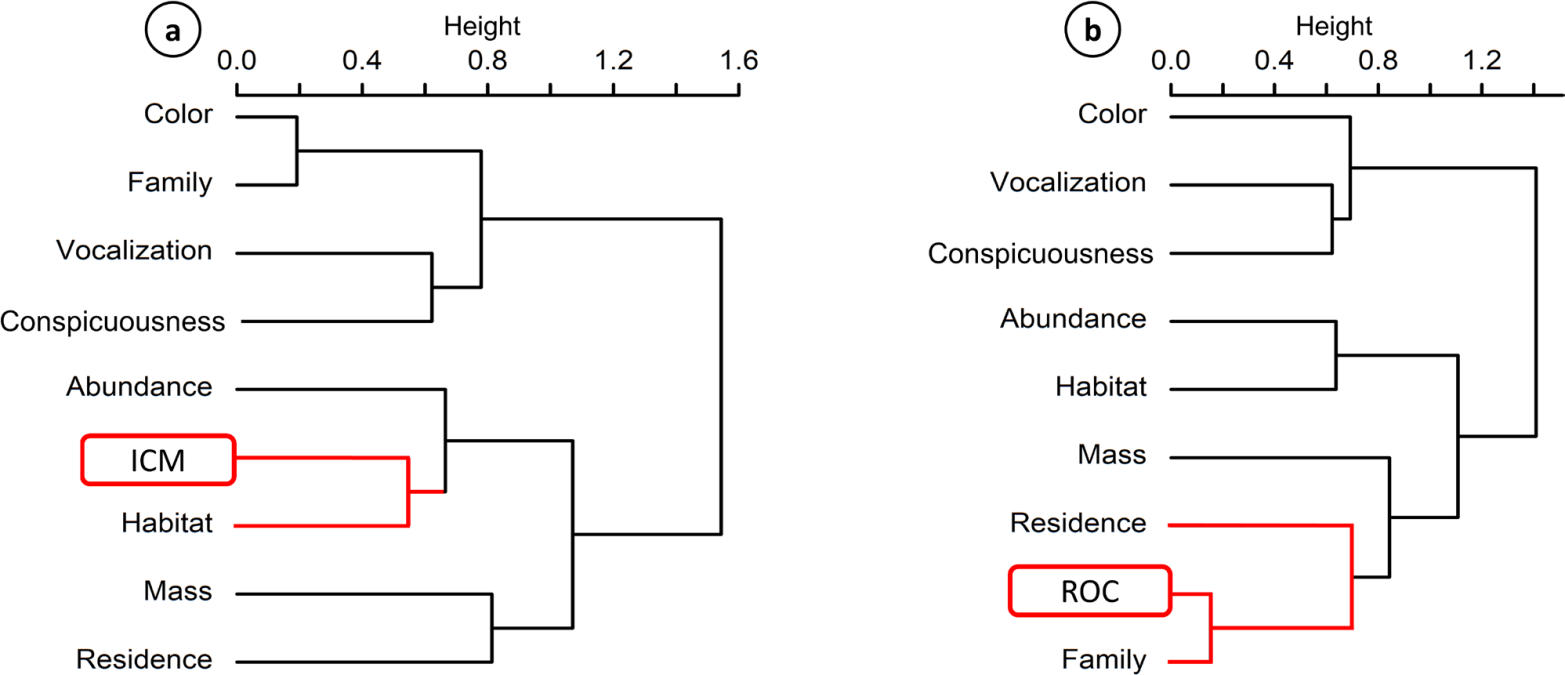
Clusters generated through hierarchical clustering analysis of the relationships between bird traits and data quality variables. Red branches indicate statistically significant associations between specific traits and data quality variables. (a) ICM, identified by community monitors (b) ROC, ratio of observed counts. Residence, residency status of the species.

Hierarchical clustering analysis revealed an association between the variable “Identified by community monitors” and the biological traits Habitat status and Abundance (Figure 5a). In particular, a statistically significant association was found between the ability of community monitors to identify species and Habitat status (*χ* ^2^ = 20.585, df = 2, *p* < 0.001), with a Cramér's *V* coefficient of 0.454, indicating a moderate to strong relationship (Cohen 1988). In contrast, Abundance did not show significant differences across categories of the “Identified by community monitors” variable (ANOVA: *F*_1,98_ = 3.20, *p* = 0.08), explaining only 3.1% of the observed variation (*η* ^2^ = 0.031).

Meanwhile, the variable “Ratio of observed counts” clustered with the trait's Taxonomic family and Residency status (Figure 5b). In this context, the accuracy of community monitors in recording bird abundances varied significantly across taxonomic families (ANOVA: *F*_32,67_ = 5.311, *p* < 0.001), with a partial eta squared of 0.717, indicating that this trait explained 71.7% of the variability in the data. Similarly, Residency status also showed a significant effect (ANOVA: *F*_1,98_ = 12.85, *p* < 0.001), explaining 11.6% of the variation in the ratio of observed counts (*η* ^2^ = 0.116).

Based on this information, categories within the *Taxonomic Family* and *Residency Status* traits that might introduce biases in the community monitoring data were selected. Within the *Taxonomic Family* trait, the families Trochilidae and Tyrannidae were selected. For this selection, the abundance recorded at the family level by both sampling groups was analyzed, and quartiles were calculated for each dataset, dividing each series into four ranges. Each range was then assigned one of the four abundance categories proposed by Howell and Webb (1995): rare (Q1), uncommon (Q2), common (Q3), and abundant (Q4). This approach allowed for the identification of specific discrepancies in which community monitors underestimated the abundance of certain taxonomic families compared to the data recorded by ornithologists. In the case of the Trochilidae family, it was classified as common by ornithologists (68 individuals), but as rare by community monitors (1 individual). Similarly, the Tyrannidae family was recorded as abundant by ornithologists (552 individuals) and as common by monitors (29 individuals). Regarding the *Residency Status* trait, migratory species were selected because they are associated with lower local familiarity, given that they are present during only part of their annual cycle (Ortega‐Álvarez and Casas 2023). Particularly, wintering migratory species tend to be less conspicuous, as they are not in their reproductive season, which results in lower vocal activity, absence of nesting behavior, and less colorful plumage (Berlanga et al. 2008; Ortega‐Álvarez and Casas 2023). Only trait categories related to the variable “Ratio of observed counts*”* were selected, as analyzing the relationship between Habitat status and the variable “Identified by community monitors” would be redundant when comparing estimates of species richness and community structure across habitat types.

Once the species belonging to the families Trochilidae and Tyrannidae, as well as migratory species, were removed from the bird lists, significant differences in species richness estimates were observed only for the forest habitat. In this habitat, the ornithologists estimated a higher species richness than the community monitors (41 ± 3 species vs. 33 ± 4 species, respectively), with nonoverlapping confidence intervals (Appendix S10). Pointwise comparisons showed that expected richness estimates were consistently greater in the ornithologists' data at all evaluated points (*Z* > 2.59, *p* < 0.01; Appendix S8). In contrast, no significant differences were observed in the anthropized habitat. Here, species richness reported by ornithologists (37 ± 3 species) and community monitors (33 ± 3 species) showed overlapping confidence intervals (Appendix S10). In pointwise comparisons, although the first evaluated points (*m* = 111 and *m* = 222) yielded *Z* values of 1.14 to 1.23, *p* values were > 0.21 (Appendix S8). At subsequent points, the magnitude of the difference remained low (*Z* < 1.0, *p* > 0.3).

Regarding bird community structure, significant differences (84% CI; Table 2) were detected only in the estimation of effective diversity for common species (*q* = 1) within the forest habitat. In this case, ornithologists reported higher diversity (22.34) compared to community monitors (18.98). However, these differences diminished when considering dominant species (*q* = 2), with similar estimates between ornithologists (15.46) and community monitors (14.25), and overlapping confidence intervals (Table 2). Likewise, the values for point dominance (Berger–Parker index = 0.140) and abundance inequality (Gini index = 0.596) recorded by ornithologists were very similar to those obtained by community monitors (Berger–Parker index = 0.152; Gini index = 0.609). In the anthropized habitat, no relevant differences were observed between ornithologists and community monitors. Effective diversity for common species (*q* = 1) was 15.47 in ornithologist data and 15.11 in monitor data; for dominant species (*q* = 2), estimates were 11.23 and 10.99, respectively (Table 2), with overlapping 84% confidence intervals in both cases. Similarly, point dominance values (Berger–Parker index: 0.171 for ornithologists vs. 0.181 for monitors) and abundance inequality (Gini index: 0.704 vs. 0.709, respectively) were nearly identical. Finally, no significant differences were found in the slopes of rank–abundance curves between data sources, either in the forest habitat (*F*_1,110_ = 1.30, *p* > 0.1) or the anthropogenic habitat (*F*_1,110_ = 2.30, *p* > 0.1; Appendix S10).

## 4 Discussion

Data collected by community monitors and ornithologists showed consistent ecological patterns when comparing the species richness and community structure of birds between forested and anthropized habitats. Consequently, the data generated by the community monitors proved useful for detecting both a decline in species richness and an increase in dominance within the bird communities of the anthropized habitat. These patterns have been well documented as typical responses of bird communities to habitat loss across different tropical vegetation types (Fahrig 2003; Sigel et al. 2010; MacGregor‐Fors and Schoundube 2011; Blandón et al. 2016; Álvarez‐Álvarez et al. 2018). However, biases were detected in the data produced by community monitors compared with those collected by ornithologists, indicating a lower efficiency in identifying species that are restricted to forest habitats. Additionally, community monitors generated lower abundance estimates for species belonging to the families Trochilidae and Tyrannidae, as well as migratory species. These biases led to an underestimation of both species' richness and the effective diversity of common species in forested habitats and influenced the characterization of community structures by producing a more pronounced dominance effect, particularly in the anthropized habitat.

Identifying these biases is crucial for making decisions aimed at the conservation and sustainable management of local habitats based on community monitoring data (Lewandowski and Specht 2015; Kosmala et al. 2016). If the species diversity associated with forest habitats is underestimated, there is a risk of undervaluing the conservation importance of primary forest areas. In this context, community monitoring data may omit species that are vulnerable to habitat loss, which is the main threat to bird conservation in the Neotropics (García‐Moreno et al. 2007). Species such as *C. guatemalensis* and *M. seductus* depend on large cavities for nesting—a resource provided exclusively by large trees typically found in primary subdeciduous tropical forests (Vázquez‐Reyes et al. 2022; Bonaparte et al. 2024). Moreover, the loss of conserved forests particularly threatens species with restricted distributions, such as *M. seductus* and *C. jocosus* , which are emblematic of the region's endemic identity (Vázquez‐Reyes et al. 2018). The community monitors also failed to record *P. ciris* , a species threatened by deforestation and significant for informing regional conservation planning (Vázquez‐Reyes et al. 2018). Biases in species identification within forest habitats may be related to the limited experience of community monitors in complex ecological settings, where factors such as dense vegetation, heterogeneous lighting, and challenging acoustic conditions hinder accurate detection, even of conspicuous species (Farr et al. 2023). In this context, some studies have shown that identification accuracy improves significantly with ongoing training and practical field experience (Lewandowski and Specht 2015; Kosmala et al. 2016). Our results align with this evidence: accumulated sampling effort had a positive and significant effect on the ability of community monitors to identify species and record abundances more accurately. These findings underscore the importance of strengthening training and field‐based learning components within community monitoring programs in Latin America.

Community monitoring data also omitted taxonomic families associated with live fences of Guamúchil ( *Pithecellobium dulce* ) located in orchards within the anthropized area. Guamúchil trees are maintained as part of agroforestry practices that provide important supplementary resources for birds. These resources can increase foraging activity among species belonging to the families Trochilidae, Tyrannidae, and particularly migratory species from the family Parulidae, potentially favoring their presence in human‐dominated areas (MacGregor‐Fors and Schoundube 2011; Ortega‐Álvarez et al. 2022). In this sense, agroforestry practices such as Guamúchil live fences contribute to increased habitat heterogeneity and resource diversity, helping to reduce the dominance effect typically observed in bird communities within anthropized systems (MacGregor‐Fors and Schoundube 2011; Ortega‐Álvarez et al. 2022). Moreover, the fruit of the Guamúchil tree constitutes a valuable medicinal and nutritional resource for indigenous communities associated with the region's tropical deciduous forest (Monroy and Colín 2004; Alcántara‐Jiménez et al. 2021; Ortega‐Álvarez et al. 2022). This information is critical for identifying and guiding sustainable agroforestry practices that simultaneously promote avian conservation and the well‐being of local communities.

A limited representation of migratory species was detected in the community monitoring data. Obtaining robust ecological data on these species is crucial for guiding management and conservation plans for the diverse ecosystems they inhabit (Hutto 2010; Vázquez‐Reyes et al. 2018). In particular, the tropical deciduous forests of the Mexican Pacific constitute key sites for the conservation of migratory birds, as they reach very high population densities during winter and occupy a wide range of habitats, making them a significant component of regional diversity (Hutto 2010). In this context, the monitoring data could be used to detect and protect resting and foraging habitats of migratory species, analyze population trends, assess the impact of human activities on migratory species, and identify local agroforestry practices that promote their diversity in both forest and anthropized habitats (McDermott et al. 2015).

In accordance with the objectives of community monitoring, the evaluation framework employed in this study addresses key aspects that, through adaptive training protocols, could maximize the quality and potential utility of data collected through projects of this nature. According to our results, these protocols could focus on improving the sampling of species that are restricted to forest habitats. They could also include activities aimed at the detection, differentiation, and counting of bird species belonging to the families Trochilidae and Tyrannidae, as well as migratory species. Similarly, by identifying species for which the data are highly accurate, it becomes possible to enrich the monitoring objectives and foster the development of projects that benefit local communities (Ortega‐Álvarez et al. 2015; Ortega‐Álvarez and Calderón‐Parra 2021). Among these projects are the creation of environmental management units (Espino‐Barros et al. 2008), payment for environmental services (Cranford and Mourato 2011), agroforestry systems (Vallejo et al. 2015); landscape restoration (Garzón et al. 2020), and avitourism (Lozada‐Ronquillo 2017). For example, in our study, the abundance data of *Eupsittula canicularis* (*Orange‐fronted Parakeet*) reported by community monitors were very similar to those recorded by ornithologists. This may be attributable to the extensive local knowledge generated about this species through the practices of capturing and trading specimens, which in the past provided economic benefits for some community monitors (Cantú et al. 2021). Given that it is a globally vulnerable species (BirdLife International 2024b) and that community monitors were able to accurately quantify its abundance, monitoring objectives focused on evaluating the status of this population would be feasible with the community monitors' current abilities (Ortega‐Álvarez and Calderón‐Parra 2021). Having this information would enable the creation of economically productive initiatives directed toward its conservation, such as avitourism (Ortega‐Álvarez and Calderón‐Parra 2021).

Additionally, beyond evaluating the quality of the monitoring data, the use of ecological parameters allowed us to explore their potential and identify possible practical limitations. In this study, the community monitoring data exhibited sufficient quality to document general patterns of ecological changes in taxonomic richness and community structure of birds in response to anthropogenic impacts. Identifying pronounced patterns of human‐induced disturbance in ecosystems is an objective that generally does not require highly rigorous accuracy in ecological data (Firmiano et al. 2017; Kosmala et al. 2016; Sgarbi et al. 2020). However, community monitoring data may be insufficient for detecting subtle gradients of anthropization or for estimating functional diversity indices due to the systematic bias associated with species restricted to forest habitats, specific taxonomic families, and migratory birds (Leitão et al. 2016; Sgarbi et al. 2020). This phenomenon is further exacerbated when the species contributing to the bias disproportionately have uncommon traits or are highly sensitive to anthropogenic impacts (Leitão et al. 2016; Firmiano et al. 2017). Indeed, this was the case in our analysis, since the birds that introduced bias in the community monitors' data significantly increased community evenness in anthropized areas. Our results confirm that validating data through the estimation of ecological parameters is a necessary practice to integrate into participatory projects if they intend to address more specialized ecological questions (Crall et al. 2010; Kosmala et al. 2016). It is also important to emphasize that the analytical limitations associated with the detected biases do not imply that community monitoring activities are ineffective for generating useful data for ecological assessments. On the contrary, they underscore the need to strengthen local capacities in bird identification and counting before employing community‐generated data in analyses aimed at addressing socioecological challenges (Conrad and Hilchey 2011; Thomsen et al. 2024).

We acknowledge that by focusing on comparisons between sampling groups (i.e., community monitors and ornithologists), our study may have overlooked observer‐specific biases related to individual skills (Kosmala et al. 2016). For instance, the ornithologists' data might be subject to biases arising from professional standards or different levels of experience in bird recording (LeResche and Rausch 1974; Ninio et al. 2003; Swanson et al. 2016). Likewise, community monitoring data could be linked to biases stemming from participants' age, occupation, and interests regarding local birds (Wyndham 2010; Ortega‐Álvarez and Casas 2023). In both groups, a key factor to consider is imperfect detection—that is, the likelihood that not all species present during sampling are recorded (Kéry and Schmidt 2008). Detection probability can be influenced by multiple factors, including species traits (e.g., vocal detectability or cryptic behavior), site‐specific environmental and biophysical conditions, and the observer's individual skills. These sources of variation can introduce biases and increase uncertainty in the collected data (Rigby and Johnson 2025). In this study, we implemented simultaneous sampling to minimize variation in bird detection probability attributable to spatial, temporal, and climatic factors. Nevertheless, we acknowledge that variability associated with individual skill levels among sampling participants remains a significant source of uncertainty (Kéry and Schmidt 2008). Future studies should address variability within the groups we evaluated (Bergstedt et al. 2009; Kosmala et al. 2016). In particular, identifying the consistency of each community monitor's performance could inform targeted training strategies aimed at fostering more homogeneous skill development across the group. A promising approach in this regard is to promote self‐training activities, where the most skilled and motivated monitors take an active role in training their peers (Ortega‐Álvarez et al. 2015). These activities not only promote collaborative learning but also strengthen community monitors' sense of ownership and autonomy in project management, while contributing to the development of more equitable capacities within the group (Ortega‐Álvarez et al. 2015). In this context, we observed feedback processes among community monitors, whereby species identification errors made by one participant were corrected by others, thus preventing misidentified species from being recorded. While this dynamic enhanced data quality by minimizing the inclusion of incorrect identifications, it also introduces an important methodological limitation in our study: It was not possible to systematically document which species were confused or how many potential errors were corrected prior to data recording. The absence of information on misidentifications (even if they were successfully resolved) limits our ability to more precisely assess the frequency and nature of such errors and, consequently, to estimate their potential impact on community monitoring data. Incorporating this dimension into future research would not only help refine training protocols but also support the development of more robust metrics for evaluating data quality in citizen science schemes. Moreover, analyzing patterns of species misidentification could help identify taxonomic groups or ecological contexts that require greater technical support, thereby improving efforts in ongoing training and data validation.

We consider that our approach offers a valuable perspective for evaluating and improving the quality of monitoring data from initiatives in other Latin American communities. In contrast to participatory projects associated with the urban context of the Global North, we argue that quality evaluation should not be limited to discarding or adapting data to address specific ecological questions. Instead, this evaluation could be integrated into a dynamic process focused on consolidating participants' ability to generate high‐quality ecological data while progressively addressing the community's research needs. In this context, scientific efforts transcend mere data validation and promote close collaboration with local communities to build and strengthen community capacity in biological monitoring. This process includes identifying species that influence data quality, defining adaptive training protocols to mitigate biases, and validating the data through the estimation of ecological parameters. Finally, identifying species for which the data are highly accurate will help enrich monitoring objectives and foster the development of conservation strategies that simultaneously address the economic needs of local communities, such as avitourism.

In the context of our study area, the Balsas River region, strengthening local capacities through community‐based monitoring training positions local communities as central actors in the conservation of their environment. This active participation not only facilitates the generation of high‐quality ecological data but also promotes initiatives aimed at habitat preservation and sustainable productive development for the benefit of the local population. However, the region lacks effective legal mechanisms to protect forest cover from land‐use change and, despite meeting the criteria to be designated as a protected natural area, has received minimal governmental support (Bezaury 2010; Vázquez‐Reyes et al. 2018). Given its high biodiversity, significant endemism (Vázquez‐Reyes et al. 2018), the existing body of ecological knowledge (Vázquez‐Reyes et al. 2017, 2022), and the growing community commitment to conservation activities linked to avitourism (Mendoza‐Lozana 2022), it is essential to establish a formal pathway toward the official protection of the territory. In this context, community‐based monitoring emerges as a strategic tool to consolidate participatory protection schemes, in which communities, beyond producing relevant information, take on an active role in the stewardship and management of their biocultural resources. Thus, the Alto Balsas region of Guerrero represents a key area for directing public investment to support these processes through an approach grounded in participatory conservation and territorial justice.

## Author Contributions

**Alexis Mendoza‐Lozana:** conceptualization (equal), data curation (lead), formal analysis (lead), investigation (lead), methodology (lead), visualization (lead), writing – original draft (lead), writing – review and editing (equal). **Rubén Ortega‐Álvarez:** conceptualization (supporting), formal analysis (supporting), methodology (supporting), supervision (equal), writing – original draft (supporting), writing – review and editing (equal). **Adolfo G. Navarro‐Sigüenza:** conceptualization (supporting), supervision (equal), writing – review and editing (equal). **Víctor H. Jiménez‐Arcos:** conceptualization (supporting), supervision (equal), writing – review and editing (equal). **Leopoldo D. Vázquez‐Reyes:** conceptualization (equal), formal analysis (supporting), funding acquisition (lead), investigation (supporting), methodology (supporting), project administration (lead), writing – original draft (supporting), writing – review and editing (equal).

## Conflicts of Interest

The authors declare no conflicts of interest.

## Supporting information


**Appendix S1:** MonitoringData.

**Appendix S2:** Script_Rarefaction.

**Appendix S3:** Bird_Data.

**Appendix S4:** Script_Cluster.

**Appendix S5:** Summary of simultaneous bird surveys conducted by ornithologists and community monitors during this study. Visit = Community visit number; Orni = Number of ornithologists participating in the surveys; Monitors = Number of community monitors participating in the surveys; Points ID = Identification codes of count points visited on that date; Temp. = Temperature range recorded during visits to count points; Total points = Total number of count points surveyed per day.

**Appendix S6:** Methodological workflow for assessing the quality of community‐based monitoring data in this study.

**Appendix S7:** Bird species recorded by ornithologists and community monitors, along with their respective biological traits. Orni = abundance recorded by ornithologists; Com = abundance recorded by community monitors; ROC = ratio of observed counts variable; ICM = identified by community monitors variable; Abun = abundance; Res = residence status; *R* = resident; M = migratory; Hab = habitat; ANTH = anthropized; FOR = forest; Cons = conspicuousness; CON = conspicuous; INCON = inconspicuous; Vocal = vocalization; MED = medium.

**Appendix S8:** Pointwise comparisons of species richness between habitat types and sampling groups, based on *Z*‐tests applied to rarefied sample sizes generated using the iNEXT R package. Max. = maximum values; Min. = minimum values.

**Appendix S9:** Improvements in data quality generated by the community monitoring group as a function of accumulated sampling effort. (a) Proportion of species identified by community monitors compared to ornithologists. (b) Ratio of individuals recorded by community monitors to those recorded by ornithologists.

**Appendix S10:** Values of species richness and community structure estimated for each habitat type by sampling group, excluding species from the families Trochilidae, Tyrannidae, and migratory birds. (a) Species richness estimated for forest habitat by both ornithologists and community monitors (462 individuals; orange dot). (b) Species richness estimated for anthropized habitat by both ornithologists and community monitors (1377 individuals; orange dot). (c) Rank–abundance curves estimated for forest habitat by both ornithologists and community monitors. (d) Rank–abundance curves estimated for anthropized habitat by both ornithologists and community monitors. Shaded area = 95% confidence intervals; IPP = individuals per point count.

## Data Availability

The code used for data analysis as well as the databases are provided as Appendices S1–, S4.
