# Supplementary material for: Does Participatory Bird Monitoring Provide Accurate Data for Ecological Research? An Experience in Rural Southwestern Mexico
Source: Ecol Evol. 2025 Oct 1;15(10):e72237. doi: 10.1002/ece3.72237 (PMC12488215; doi:10.1002/ece3.72237)
Supplement: Supplementary file 5 — Appendix S5: Summary of simultaneous bird surveys conducted by ornithologists and community monitors during this study. Visit = Community visit number; Orni = Number of ornithologists participating in the surveys; Monitors = Number of community monitors participating in the surveys; Points ID = Identification codes of count points visited on that date; Temp. = Temperature range recorded during visits to count points; Total points = Total number of count points surveyed per day. [file ECE3-15-e72237-s003.docx]

**Appendix S5. Summary of simultaneous bird surveys conducted by ornithologists and community monitors during this study.** Visit = Community visit number; Orni = Number of ornithologists participating in the surveys; Monitors = Number of community monitors participating in the surveys; Points ID = Identification codes of count points visited on that date; Temp. = Temperature range recorded during visits to count points; Total points = Total number of count points surveyed per day

| **Visit** | **Date** | **Orni** | **Monitors** | **Habitat** | **Points ID** | **Start time** | **End Time** | **Temp.** | **Cloud Cover** | **Total points** |
| --- | --- | --- | --- | --- | --- | --- | --- | --- | --- | --- |
| 1 | 2023-04-20 | 2 | 5 | Forest | F 1-7 | 06:30 | 08:52 | 19-24°C | 20% | 7 |
| 1 | 2023-04-21 | 2 | 5 | Anthropized | A1-7 | 06:27 | 08:22 | 21-25°C | 20% | 14 |
| 1 | 2023-04-22 | 2 | 5 | Forest | F8-14 | 06:42 | 09:10 | 19-25°C | 20% | 21 |
| 1 | 2023-04-23 | 2 | 5 | Anthropized | A8-14 | 06:37 | 08:15 | 19-25°C | 20% | 28 |
| 2 | 2023-06-12 | 2 | 5 | Forest | F8-14 | 07:04 | 09:20 | 18-21°C | 0% | 35 |
| 2 | 2023-06-13 | 2 | 5 | Forest | F 1-7 | 06:59 | 09:03 | 17-20°C | 0% | 42 |
| 2 | 2023-06-14 | 2 | 5 | Anthropized | A1-7 | 07:16 | 08:45 | 17-20°C | 10% | 49 |
| 2 | 2023-06-15 | 2 | 5 | Anthropized | A8-14 | 07:05 | 08:52 | 17-20°C | 10% | 56 |
| 3 | 2023-08-26 | 1 | 4 | Anthropized | A8-14 | 06:43 | 08:47 | 18-21°C | 0% | 63 |
| 3 | 2023-08-27 | 1 | 4 | Anthropized | A1-7 | 06:31 | 08:30 | 20-23°C | 0% | 70 |
| 3 | 2023-08-28 | 2 | 4 | Forest | F 1-7 | 06:43 | 09:02 | 21-24°C | 0% | 77 |
| 3 | 2023-08-29 | 2 | 4 | Forest | F8-14 | 06:32 | 08:54 | 21-24°C | 0% | 84 |
| 4 | 2023-10-16 | 1 | 5 | Forest | F8-14 | 07:13 | 09:26 | 21-23°C | 0% | 91 |
| 4 | 2023-10-17 | 2 | 5 | Anthropized | A1-7 | 07:00 | 08:42 | 21-24°C | 0% | 98 |
| 4 | 2023-10-18 | 2 | 5 | Forest | F 1-7 | 07:05 | 09:17 | 21-24°C | 10% | 105 |
| 4 | 2023-10-19 | 2 | 4 | Anthropized | A8-14 | 07:00 | 08:40 | 21-24°C | 10% | 112 |
| 5 | 2024-01-26 | 1 | 5 | Anthropized | A1-7 | 07:03 | 08:56 | 14-17°C | 0% | 119 |
| 5 | 2024-01-27 | 2 | 5 | Anthropized | A8-14 | 07:10 | 09:00 | 14-17°C | 0% | 126 |
| 5 | 2024-01-28 | 2 | 5 | Forest | F 1-7 | 07:15 | 09:30 | 15-18°C | 0% | 133 |
| 5 | 2024-01-29 | 2 | 5 | Forest | F8-14 | 07:12 | 09:22 | 15-18°C | 0% | 140 |
| 6 | 2024-01-21 | 2 | 4 | Forest | F 1-7 | 07:01 | 09:18 | 16-19°C | 10% | 147 |
| 6 | 2024-01-22 | 2 | 5 | Forest | F8-14 | 07:00 | 09:15 | 20-23°C | 10% | 154 |
| 6 | 2024-01-23 | 2 | 5 | Anthropized | A8-14 | 07:08 | 08:56 | 18-21°C | 20% | 161 |
| 6 | 2024-01-24 | 2 | 5 | Anthropized | A1-7 | 07:00 | 09:00 | 18-21°C | 20% | 168 |
