# Supplementary material for: Does Participatory Bird Monitoring Provide Accurate Data for Ecological Research? An Experience in Rural Southwestern Mexico
Source: Ecol Evol. 2025 Oct 1;15(10):e72237. doi: 10.1002/ece3.72237 (PMC12488215; doi:10.1002/ece3.72237)

**Appendix S6. Methodological workflow for assessing the quality of community-based monitoring data in this stu****dy**


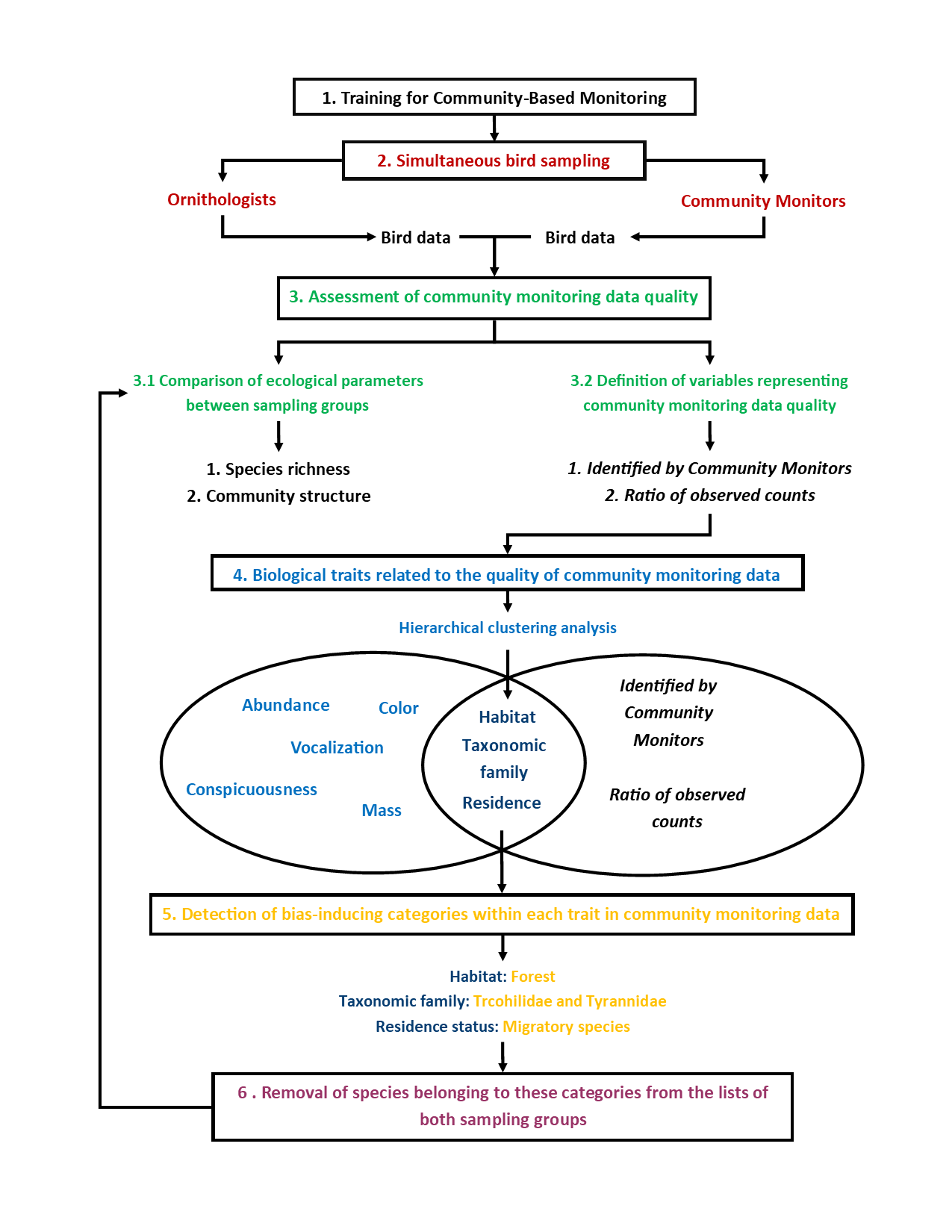

Supplement: Supplementary file 6 — Appendix S6: Methodological workflow for assessing the quality of community‐based monitoring data in this study. [file ECE3-15-e72237-s004.docx]
