# Supplementary material for: Does Participatory Bird Monitoring Provide Accurate Data for Ecological Research? An Experience in Rural Southwestern Mexico
Source: Ecol Evol. 2025 Oct 1;15(10):e72237. doi: 10.1002/ece3.72237 (PMC12488215; doi:10.1002/ece3.72237)
Supplement: Supplementary file 7 — Appendix S7: Bird species recorded by ornithologists and community monitors, along with their respective biological traits. Orni = abundance recorded by ornithologists; Com = abundance recorded by community monitors; ROC = ratio of observed counts variable; ICM = identified by community monitors variable; Abun = abundance; Res = residence status; R = resident; M = migratory; Hab = habitat; ANTH = anthropized; FOR = forest; Cons = conspicuousness; CON = conspicuous; INCON = inconspicuous; Vocal = vocalization; MED = medium. [file ECE3-15-e72237-s005.docx]

**Appendix S7. Bird species recorded by ornithologists and community monitors, along with their respective biological traits.** Orni = Abundance recorded by ornithologists; Com = Abundance recorded by community monitors; ROC = Ratio of observed counts variable; ICM = Identified by Community Monitors variable; Abun = Abundance; Res = Residence status; R = Resident; M = Migratory; Hab = Habitat; ANTH = Anthropized; FOR = Forest; Cons = Conspicuousness; CON = Conspicuous; INCON = Inconspicuous; Vocal = Vocalization; MED = Medium.

|  | | | | | | | | | | | | | | |
| --- | --- | --- | --- | --- | --- | --- | --- | --- | --- | --- | --- | --- | --- | --- |
| **Order** | **Family** | **Genus** | **Specie** | **Orni** | **Com** | **ROC** | **CM** | **Abun** | **Mass** | **Res** | **Color** | **Hab** | **Cons** | **Vocal** |
| Accipitriformes | Cracidae | Ortalis | *Ortalis poliocephala* | 22 | 20 | 0.90 | YES | 16 | 760 | R | 23.91 | BOTH | CON | HIGH |
| Galliformes | Odontophoridae | Philortyx | *Philortyx fasciatus* | 11 | 4 | 0.36 | YES | 11 | 130 | R | 22.45 | ANTH | INCON | MED |
| Columbiformes | Columbidae | Columba | *Columba livia* | 2 | 2 | 1.00 | YES | 2 | 354.2 | R | 24.96 | ANTH | CON | LOW |
| Columbiformes | Columbidae | Streptopelia | *Streptopelia decaocto* | 145 | 96 | 1.00 | YES | 145 | 149 | R | 20.22 | ANTH | CON | HIGH |
| Columbiformes | Columbidae | Columbina | *Columbina inca* | 368 | 346 | 0.94 | YES | 368 | 47.5 | R | 19.70 | BOTH | CON | MED |
| Columbiformes | Columbidae | Columbina | *Columbina passerina* | 94 | 90 | 0.95 | YES | 94 | 35.4 | R | 17.31 | BOTH | INCON | MED |
| Columbiformes | Columbidae | Leptotila | *Leptotila verreauxi* | 50 | 46 | 0.92 | YES | 50 | 146.9 | R | 25.14 | BOTH | INCON | HIGH |
| Columbiformes | Columbidae | Zenaida | *Zenaida asiatica* | 27 | 31 | 1.15 | YES | 27 | 153 | R | 18.72 | BOTH | CON | MED |
| Columbiformes | Columbidae | Zenaida | *Zenaida macroura* | 70 | 66 | 0.94 | YES | 70 | 118.9 | R | 16.20 | BOTH | INCON | MED |
| Cuculiformes | Cuculidae | Crotophaga | *Crotophaga sulcirostris* | 111 | 77 | 0.69 | YES | 111 | 82 | R | 34.42 | BOTH | CON | HIGH |
| Cuculiformes | Cuculidae | Morococcyx | *Morococcyx erythropygus* | 3 | 1 | 0.33 | YES | 3 | 64.5 | R | 21.37 | BOTH | INCON | HIGH |
| Cuculiformes | Cuculidae | Geococcyx | *Geococcyx velox* | 1 | 1 | 1.00 | YES | 1 | 179.9 | R | 33.45 | FOR | INCON | LOW |
| Cuculiformes | Cuculidae | Piaya | *Piaya cayana* | 4 | 4 | 1.00 | YES | 4 | 102 | R | 31.30 | FOR | INCON | LOW |
| Apodiformes | Trochilidae | Heliomaster | *Heliomaster constantii* | 2 | 0 | 0.00 | NO | 2 | 7.3 | R | 25.62 | FOR | CON | LOW |
| Apodiformes | Trochilidae | Archilochus | *Archilochus colubris* | 19 | 0 | 0.00 | NO | 19 | 3.1 | M | 29.54 | BOTH | CON | LOW |
| Apodiformes | Trochilidae | Archilochus | *Archilochus alexandri* | 7 | 0 | 0.00 | NO | 7 | 3.4 | M | 31.25 | BOTH | CON | LOW |
| Apodiformes | Trochilidae | Selasphorus | *Selasphorus rufus* | 8 | 0 | 0.00 | NO | 8 | 3.5 | M | 34.27 | BOTH | CON | LOW |
| Apodiformes | Trochilidae | Selasphorus | *Selasphorus platycercus* | 3 | 0 | 0.00 | NO | 3 | 3.5 | R | 30.74 | ANTH | CON | LOW |
| Apodiformes | Trochilidae | Phaeoptila | *Phaeoptila sordida* | 17 | 1 | 0.05 | YES | 17 | 10 | R | 24.39 | BOTH | CON | LOW |
| Apodiformes | Trochilidae | Ramosomyia | *Ramosomyia violiceps* | 12 | 0 | 0.00 | NO | 12 | 259.4 | R | 29.38 | BOTH | CON | LOW |
| Charadiiformes | Charadriidae | Charadrius | *Charadrius vociferus* | 2 | 2 | 1.00 | YES | 2 | 96.4 | R | 28.46 | ANTH | CON | LOW |
| Pelecaniformes | Ardeidae | Ardea | *Ardea herodias* | 2 | 1 | 0.50 | YES | 1 | 2523.4 | R | 27.16 | FOR | CON | LOW |
| Pelecaniformes | Ardeidae | Egretta | *Egretta thula* | 5 | 4 | 0.80 | YES | 5 | 371 | R | 38.18 | BOTH | CON | LOW |
| Pelecaniformes | Ardeidae | Butorides | *Butorides virescens* | 1 | 0 | 0.00 | NO | 1 | 201.5 | R | 27.09 | ANTH | CON | MED |
| Accipitriformes | Accipitridae | Buteogallus | *Buteogallus anthracinus* | 1 | 1 | 1.00 | YES | 1 | 975.1 | R | 33.64 | FOR | INCON | MED |
| Accipitriformes | Accipitridae | Buteo | *Buteo plagiatus* | 10 | 7 | 0.70 | YES | 10 | 519 | R | 25.95 | BOTH | CON | MED |
| Strigiformes | Strigidae | Megascops | *Megascops seductus* | 3 | 0 | 0.00 | NO | 3 | 160 | R | 24.38 | FOR | INCON | HIGH |
| Strigiformes | Strigidae | Glaucidium | *Glaucidium palmarum* | 6 | 0 | 0.00 | NO | 6 | 45.7 | R | 22.34 | BOTH | INCON | MED |
| Trogoniformes | Trogonidae | Trogon | *Trogon elegans* | 7 | 5 | 0.71 | YES | 7 | 70.9 | R | 33.87 | BOTH | CON | LOW |
| Coraciiformes | Momotidae | Momotus | *Momotus mexicanus* | 33 | 14 | 0.42 | YES | 33 | 75.7 | R | 28.99 | BOTH | CON | HIGH |
| Coraciiformes | Alcedinidae | Megaceryle | *Megaceryle alcyon* | 1 | 0 | 0.00 | NO | 1 | 148 | M | 26.91 | FOR | CON | LOW |
| Piciformes | Picidae | Melanerpes | *Melanerpes chrysogenys* | 152 | 109 | 0.72 | YES | 152 | 67.7 | R | 33.70 | BOTH | CON | HIGH |
| Piciformes | Picidae | Campephilus | *Campephilus guatemalensis* | 2 | 0 | 0.00 | NO | 2 | 242 | R | 37.09 | FOR | CON | MED |
| Falconiformes | Falconidae | Herpetotheres | *Herpetotheres cachinnans* | 1 | 0 | 0.00 | NO | 1 | 623.6 | R | 29.60 | ANTH | CON | HIGH |
| Falconiformes | Falconidae | Falco | *Falco sparverius* | 6 | 1 | 0.16 | YES | 6 | 114.6 | M | 30.57 | ANTH | INCON | LOW |
| Psittaciformes | Psittacidae | Eupsittula | *Eupsittula canicularis* | 41 | 52 | 1.27 | YES | 41 | 85 | R | 50.70 | BOTH | CON | HIGH |
| Passeriformes | Tyrannidae | Camptostoma | *Camptostoma imberbe* | 13 | 1 | 0.07 | YES | 13 | 7.4 | R | 31.43 | BOTH | INCON | MED |
| Passeriformes | Tyrannidae | Myiarchus | *Myiarchus tuberculifer* | 26 | 18 | 0.11 | YES | 26 | 17.7 | R | 24.15 | BOTH | INCON | MED |
| Passeriformes | Tyrannidae | Myiarchus | *Myiarchus cinerascens* | 1 | 0 | 0.00 | NO | 1 | 28.2 | M | 25.39 | ANTH | INCON | MED |
| Passeriformes | Tyrannidae | Myiarchus | *Myiarchus nuttingi* | 1 | 0 | 0.00 | NO | 1 | 23 | R | 25.52 | FOR | INCON | MED |
| Passeriformes | Tyrannidae | Myiarchus | *Myiarchus tyrannulus* | 10 | 1 | 0.10 | YES | 10 | 35.5 | R | 24.06 | BOTH | INCON | MED |
| Passeriformes | Tyrannidae | Pitangus | *Pitangus sulphuratus* | 29 | 9 | 0.30 | YES | 29 | 62.9 | R | 35.63 | BOTH | CON | HIGH |
| Passeriformes | Tyrannidae | Myiozetetes | *Myiozetetes similis* | 23 | 0 | 0.00 | NO | 23 | 28 | R | 34.01 | BOTH | CON | HIGH |
| Passeriformes | Tyrannidae | Myiodynastes | *Myiodynastes luteiventris* | 43 | 29 | 0.67 | YES | 43 | 46.9 | M | 30.61 | BOTH | CON | HIGH |
| Passeriformes | Tyrannidae | Tyrannus | *Tyrannus melancholicus* | 293 | 150 | 0.51 | YES | 293 | 37.4 | R | 29.76 | BOTH | INCON | HIGH |
| Passeriformes | Tyrannidae | Tyrannus | *Tyrannus crassirostris* | 5 | 1 | 0.20 | YES | 5 | 55.9 | R | 26.99 | ANTH | INCON | MED |
| Passeriformes | Tyrannidae | Tyrannus | *Tyrannus verticalis* | 30 | 6 | 0.20 | YES | 30 | 39.6 | M | 32.95 | BOTH | INCON | HIGH |
| Passeriformes | Tyrannidae | Contopus | *Contopus sordidulus* | 1 | 0 | 0.00 | NO | 1 | 13.1 | M | 23.47 | FOR | CON | HIGH |
| Passeriformes | Tyrannidae | Empidonax | *Empidonax minimus* | 31 | 0 | 0.00 | NO | 31 | 10 | M | 26.39 | BOTH | INCON | MED |
| Passeriformes | Tyrannidae | Empidonax | *Empidonax wrightii* | 8 | 0 | 0.00 | NO | 8 | 12.3 | M | 25.19 | BOTH | INCON | MED |
| Passeriformes | Tyrannidae | Empidonax | *Empidonax difficilis* | 20 | 0 | 0.00 | NO | 20 | 10.7 | M | 23.41 | FOR | INCON | MED |
| Passeriformes | Tyrannidae | Pyrocephalus | *Pyrocephalus rubinus* | 18 | 5 | 0.28 | YES | 18 | 14.4 | R | 34.71 | ANTH | CON | LOW |
| Passeriformes | Furnariidae | Xiphorhynchus | *Xiphorhynchus flavigaster* | 4 | 0 | 0.00 | NO | 4 | 46.2 | R | 27.23 | FOR | INCON | LOW |
| Passeriformes | Furnariidae | Lepidocolaptes | *Lepidocolaptes leucogaster* | 1 | 0 | 0.00 | NO | 1 | 36 | R | 27.60 | FOR | INCON | MED |
| Passeriformes | Vireonidae | Vireo | *Vireo gilvus* | 3 | 1 | 0.33 | YES | 3 | 12.7 | M | 26.03 | FOR | INCON | LOW |
| Passeriformes | Laniidae | Lanius | *Lanius ludovicianus* | 1 | 0 | 0.00 | NO | 1 | 51.6 | R | 33.15 | ANTH | CON | LOW |
| Passeriformes | Corvidae | Calocitta | *Calocitta formosa* | 26 | 26 | 1.00 | YES | 21 | 210 | R | 39.63 | FOR | CON | HIGH |
| Passeriformes | Hirundinidae | Stelgidopteryx | *Stelgidopteryx serripennis* | 264 | 112 | 0.42 | YES | 264 | 15.7 | R | 23.04 | BOTH | CON | LOW |
| Passeriformes | Hirundinidae | Hirundo | *Hirundo rustica* | 98 | 65 | 0.66 | YES | 65 | 17.9 | M | 28.73 | ANTH | CON | LOW |
| Passeriformes | Hirundinidae | Petrochelidon | *Petrochelidon pyrrhonota* | 76 | 0 | 0.00 | NO | 76 | 21.6 | M | 25.27 | ANTH | INCON | LOW |
| Passeriformes | Polioptilidae | Polioptila | *Polioptila caerulea* | 107 | 25 | 0.23 | YES | 107 | 5.8 | M | 32.07 | BOTH | CON | HIGH |
| Passeriformes | Polioptilidae | Polioptila | *Polioptila albiloris* | 6 | 0 | 0.00 | NO | 6 | 6.3 | R | 33.44 | FOR | INCON | MED |
| Passeriformes | Troglodytidae | Catherpes | *Catherpes mexicanus* | 6 | 0 | 0.00 | NO | 6 | 12 | R | 25.29 | FOR | CON | HIGH |
| Passeriformes | Troglodytidae | Pheugopedius | *Pheugopedius felix* | 7 | 0 | 0.00 | NO | 18 | 12.8 | R | 28.99 | FOR | INCON | HIGH |
| Passeriformes | Troglodytidae | Thryophilus | *Thryophilus sinaloa* | 6 | 0 | 0.00 | NO | 2 | 15.1 | R | 25.43 | FOR | INCON | HIGH |
| Passeriformes | Troglodytidae | Thryophilus | *Thryophilus pleurostictus* | 49 | 14 | 0.29 | YES | 49 | 17.7 | R | 28.29 | FOR | INCON | HIGH |
| Passeriformes | Troglodytidae | Campylorhynchus | *Campylorhynchus jocosus* | 3 | 0 | 0.00 | NO | 3 | 27.6 | R | 30.32 | FOR | CON | HIGH |
| Passeriformes | Mimidae | Toxostoma | *Toxostoma curvirostre* | 2 | 2 | 1.00 | YES | 2 | 80.5 | R | 17.69 | ANTH | INCON | MED |
| Passeriformes | Turdidae | Turdus | *Turdus rufopalliatus* | 109 | 77 | 0.71 | YES | 109 | 75 | R | 24.60 | BOTH | CON | HIGH |
| Passeriformes | Passeridae | Passer | *Passer domesticus* | 187 | 143 | 0.77 | YES | 187 | 26.5 | R | 33.04 | ANTH | CON | HIGH |
| Passeriformes | Fringillidae | Haemorhous | *Haemorhous mexicanus* | 104 | 69 | 0.66 | YES | 104 | 21.4 | R | 18.60 | BOTH | CON | HIGH |
| Passeriformes | Fringillidae | Spinus | *Spinus psaltria* | 5 | 0 | 0.00 | NO | 5 | 8.8 | R | 35.57 | ANTH | CON | MED |
| Passeriformes | Passerellidae | Peucaea | *Peucaea ruficauda* | 74 | 20 | 0.09 | YES | 74 | 28.2 | R | 29.54 | BOTH | INCON | MED |
| Passeriformes | Passerellidae | Peucaea | *Peucaea humeralis* | 11 | 1 | 0.09 | YES | 11 | 23.9 | R | 24.43 | BOTH | CON | MED |
| Passeriformes | Passerellidae | Chondestes | *Chondestes grammacus* | 11 | 0 | 0.00 | NO | 11 | 29 | M | 25.03 | ANTH | CON | LOW |
| Passeriformes | Icteriidae | Icteria | *Icteria virens* | 3 | 1 | 0.33 | YES | 3 | 24.9 | M | 29.06 | ANTH | INCON | LOW |
| Passeriformes | Icteridae | Cassiculus | *Cassiculus melanicterus* | 1 | 0 | 0.00 | NO | 1 | 81.3 | R | 40.78 | ANTH | CON | LOW |
| Passeriformes | Icteridae | Icterus | *Icterus wagleri* | 16 | 7 | 0.89 | YES | 16 | 41.8 | R | 37.76 | BOTH | CON | HIGH |
| Passeriformes | Icteridae | Icterus | *Icterus spurius* | 26 | 23 | 0.89 | YES | 26 | 19.4 | M | 34.54 | BOTH | CON | HIGH |
| Passeriformes | Icteridae | Icterus | *Icterus pustulatus* | 125 | 80 | 0.89 | YES | 125 | 36.8 | R | 47.96 | BOTH | CON | HIGH |
| Passeriformes | Icteridae | Icterus | *Icterus gularis* | 1 | 0 | 0.00 | NO | 1 | 55.2 | R | 47.05 | ANTH | CON | HIGH |
| Passeriformes | Icteridae | Molothrus | *Molothrus aeneus* | 267 | 213 | 0.80 | YES | 267 | 62.6 | R | 33.01 | BOTH | CON | LOW |
| Passeriformes | Icteridae | Quiscalus | *Quiscalus mexicanus* | 49 | 37 | 0.76 | YES | 49 | 160.5 | R | 34.14 | ANTH | CON | HIGH |
| Passeriformes | Parulidae | Parkesia | *Parkesia noveboracensis* | 1 | 0 | 0.00 | NO | 1 | 16.3 | M | 21.47 | FOR | INCON | LOW |
| Passeriformes | Parulidae | Mniotilta | *Mniotilta varia* | 1 | 0 | 0.00 | NO | 1 | 10.9 | M | 31.60 | ANTH | CON | LOW |
| Passeriformes | Parulidae | Leiothlypis | *Leiothlypis ruficapilla* | 5 | 0 | 0.00 | NO | 5 | 8.1 | M | 32.37 | FOR | CON | LOW |
| Passeriformes | Parulidae | Leiothlypis | *Leiothlypis virginiae* | 1 | 0 | 0.00 | NO | 1 | 8.2 | M | 25.58 | FOR | CON | LOW |
| Passeriformes | Parulidae | Setophaga | *Setophaga petechia* | 7 | 0 | 0.00 | NO | 7 | 10.2 | M | 46.02 | ANTH | CON | MED |
| Passeriformes | Parulidae | Setophaga | *Setophaga coronata* | 14 | 2 | 0.14 | YES | 3 | 11.9 | M | 29.91 | BOTH | CON | LOW |
| Passeriformes | Parulidae | Setophaga | *Setophaga nigrescens* | 2 | 0 | 0.00 | NO | 2 | 8.7 | M | 31.83 | ANTH | INCON | MED |
| Passeriformes | Parulidae | Cardellina | *Cardellina pusilla* | 3 | 1 | 0.33 | YES | 3 | 7 | M | 42.41 | FOR | CON | LOW |
| Passeriformes | Cardinalidae | Piranga | *Piranga ludoviciana* | 5 | 0 | 0.00 | NO | 5 | 28.1 | M | 35.15 | BOTH | CON | LOW |
| Passeriformes | Cardinalidae | Pheucticus | *Pheucticus chrysopeplus* | 1 | 1 | 1.00 | YES | 1 | 77.6 | R | 46.16 | FOR | CON | LOW |
| Passeriformes | Cardinalidae | Pheucticus | *Pheucticus melanocephalus* | 4 | 4 | 1.00 | YES | 4 | 47.1 | R | 35.11 | BOTH | CON | LOW |
| Passeriformes | Cardinalidae | Passerina | *Passerina caerulea* | 1 | 0 | 0.00 | NO | 1 | 27.4 | R | 43.96 | FOR | CON | LOW |
| Passeriformes | Cardinalidae | Passerina | *Passerina leclancherii* | 37 | 27 | 0.73 | YES | 37 | 14 | R | 38.09 | FOR | CON | LOW |
| Passeriformes | Cardinalidae | Passerina | *Passerina versicolor* | 13 | 7 | 0.54 | YES | 13 | 12.9 | R | 36.08 | BOTH | INCON | LOW |
| Passeriformes | Cardinalidae | Passerina | *Passerina ciris* | 1 | 0 | 0.00 | NO | 1 | 15.5 | M | 39.15 | FOR | CON | MED |
| Passeriformes | Thraupidae | Volatinia | *Volatinia jacarina* | 30 | 9 | 0.30 | YES | 30 | 9.9 | R | 27.61 | ANTH | INCON | MED |
| Passeriformes | Thraupidae | Sporophila | *Sporophila torqueola* | 14 | 9 | 0.64 | YES | 14 | 8.7 | R | 30.35 | BOTH | CON | LOW |
