# Supplementary material for: Does Participatory Bird Monitoring Provide Accurate Data for Ecological Research? An Experience in Rural Southwestern Mexico
Source: Ecol Evol. 2025 Oct 1;15(10):e72237. doi: 10.1002/ece3.72237 (PMC12488215; doi:10.1002/ece3.72237)
Supplement: Supplementary file 8 — Appendix S8: Pointwise comparisons of species richness between habitat types and sampling groups, based on Z‐tests applied to rarefied sample sizes generated using the iNEXT R package. Max. = maximum values; Min. = minimum values. [file ECE3-15-e72237-s006.docx]

| **Appendix S8. Pointwise comparisons of species richness between habitat types and sampling groups, based on Z-tests applied to rarefied sample sizes generated using the iNEXT R package.** Max. = Maximum values; Min. = Minimum values. | | | | | | |
| --- | --- | --- | --- | --- | --- | --- |
| **Comparison** | **Range of ample size (m)** | **Max. Z** | **Min. Z** | **Max. *p*** | **Min. *p*** | **Significance** |
| Ornithologists: Forest vs Anthropized | 49 – 879 | 8.47 | 4.46 | < 0.00001 | < 0.00001 | YES |
| Community monitors: Forest vs Anthropized | 33 – 581 | 26.45 | 8.79 | < 0.00001 | < 0.00001 | YES |
| Forest habitat: Ornithologists vs Community monitors | 33 – 581 | 8.94 | 4.46 | < 0.00001 | < 0.00001 | YES |
| Anthropized habitat: Ornithologists vs Community monitors | 151 – 1470 | 10.50 | 8.79 | < 0.00001 | < 0.00001 | YES |
|  |  |  |  |  |  |  |
| **Comparisons of species richness excluding species from the families Trochilidae and Tyrannidae, as well as migratory species** | | | | | | |
| Forest habitat: Ornithologists vs Community monitors | 30 – 462 | 3.68 | 2.59 | < 0.001 | < 0.0001 | YES |
| Anthropized habitat: Ornithologists vs Community monitors | 111 – 1377 | 3.60 | 1.24 | 0.21 | < 0.001 | NO |
