# Supplementary material for: Does Participatory Bird Monitoring Provide Accurate Data for Ecological Research? An Experience in Rural Southwestern Mexico
Source: Ecol Evol. 2025 Oct 1;15(10):e72237. doi: 10.1002/ece3.72237 (PMC12488215; doi:10.1002/ece3.72237)

**Appendix S9. Improvements in data quality generated by the community monitoring group as a function of accumulated sampling effort.** a) Proportion of species identified by community monitors compared to ornithologists. b) Ratio of individuals recorded by community monitors to those recorded by ornithologists.


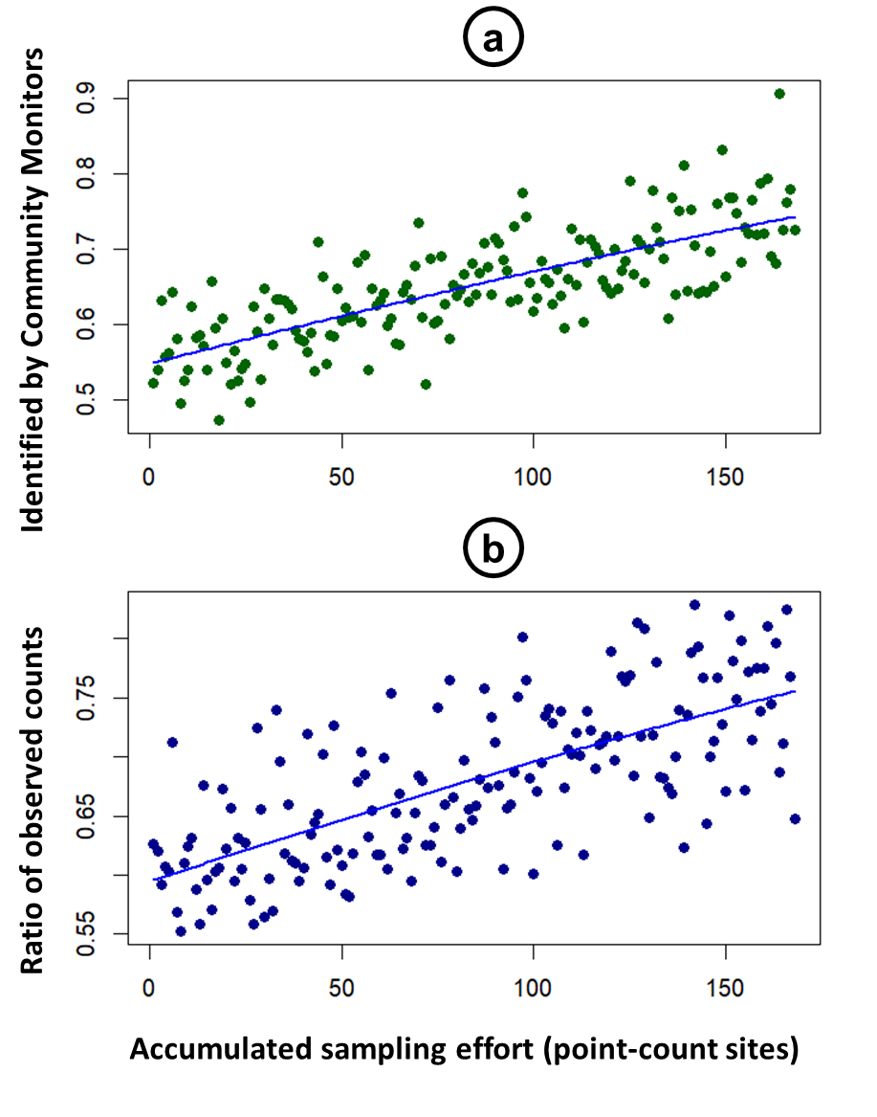

Supplement: Supplementary file 9 — Appendix S9: Improvements in data quality generated by the community monitoring group as a function of accumulated sampling effort. (a) Proportion of species identified by community monitors compared to ornithologists. (b) Ratio of individuals recorded by community monitors to those recorded by ornithologists. [file ECE3-15-e72237-s009.docx]
