# Supplementary material for: Does Participatory Bird Monitoring Provide Accurate Data for Ecological Research? An Experience in Rural Southwestern Mexico
Source: Ecol Evol. 2025 Oct 1;15(10):e72237. doi: 10.1002/ece3.72237 (PMC12488215; doi:10.1002/ece3.72237)

**Appendix S10. Values of species richness and community structure estimated for each habitat type by sampling group, excluding species from the families Trochilidae, Tyrannidae, and migratory birds.** a) Species richness estimated for forest habitat by both ornithologists and community monitors (462 individuals; orange dot). b) Species richness estimated for anthropized habitat by both ornithologists and community monitors (1377 individuals; orange dot). c) Rank-abundance curves estimated for forest habitat by both ornithologists and community monitors. d) Rank-abundance curves estimated for anthropized habitat by both ornithologists and community monitors. Shaded area = 95% confidence intervals; IPP = individuals per point count.


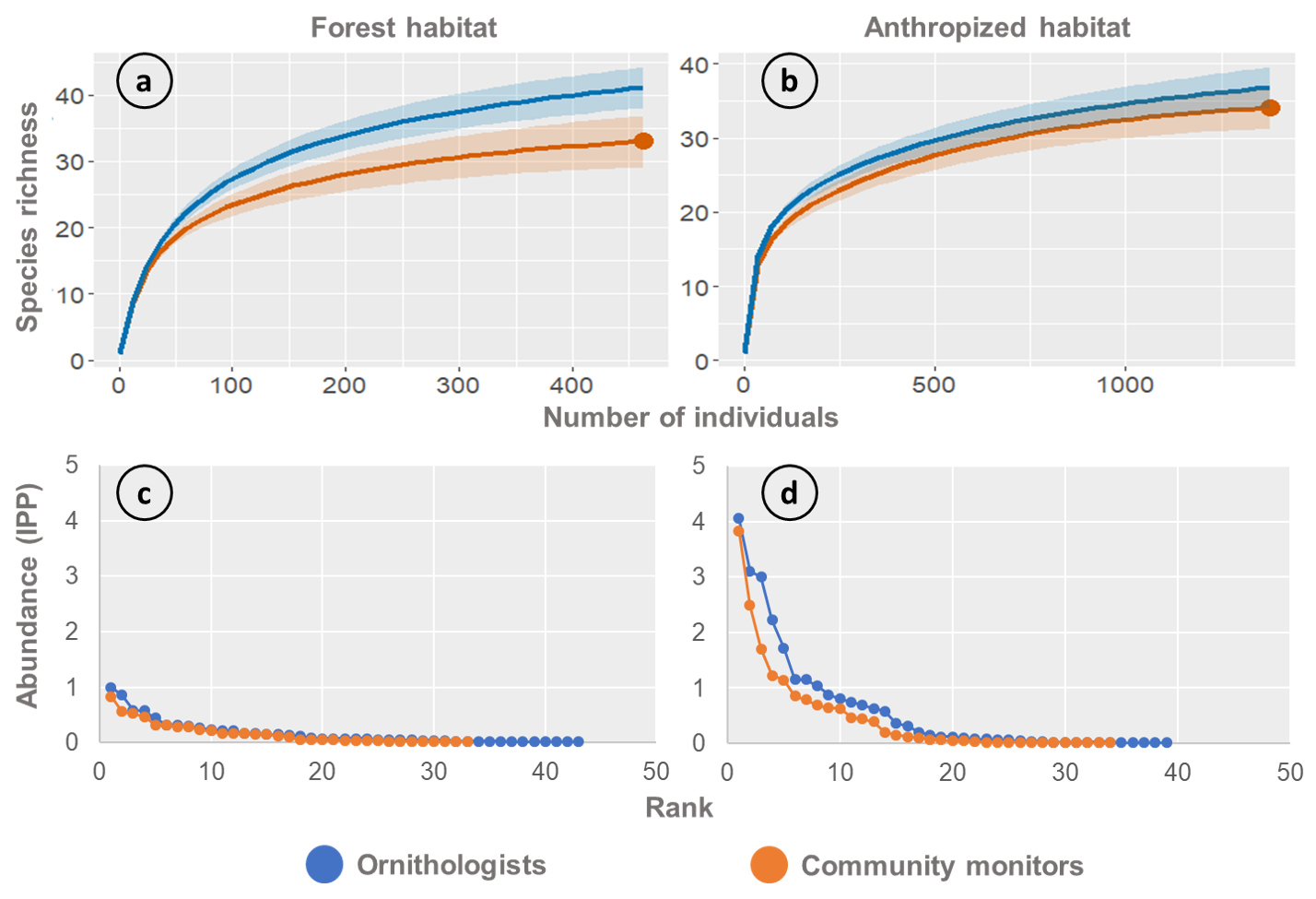

Supplement: Supplementary file 10 — Appendix S10: Values of species richness and community structure estimated for each habitat type by sampling group, excluding species from the families Trochilidae, Tyrannidae, and migratory birds. (a) Species richness estimated for forest habitat by both ornithologists and community monitors (462 individuals; orange dot). (b) Species richness estimated for anthropized habitat by both ornithologists and community monitors (1377 individuals; orange dot). (c) Rank–abundance curves estimated for forest habitat by both ornithologists and community monitors. (d) Rank–abundance curves estimated for anthropized habitat by both ornithologists and community monitors. Shaded area = 95% confidence intervals; IPP = individuals per point count. [file ECE3-15-e72237-s001.docx]
